# Gene Expression Profile of Adult Human Olfactory Bulb and Embryonic Neural Stem Cell Suggests Distinct Signaling Pathways and Epigenetic Control

**DOI:** 10.1371/journal.pone.0033542

**Published:** 2012-04-02

**Authors:** Hany E. S. Marei, Abd-Elmaksoud Ahmed, Fabrizio Michetti, Mario Pescatori, Roberto Pallini, Patricia Casalbore, Carlo Cenciarelli, Mohamed Elhadidy

**Affiliations:** 1 Department of Cytology and Histology, Faculty of Veterinary Medicine, Mansoura University, Mansoura, Egypt; 2 Department of Bacteriology, Faculty of Veterinary Medicine, Mansoura University, Mansoura, Egypt; 3 Institute of Anatomy and Cell Biology, Università Cattolica del S. Cuore, Roma, Italy; 4 Institute of Neurosurgery, Università Cattolica del Sacro Cuore, Roma, Italy; 5 Institute of Cell Biology and Neurobiology, National Research Council of Italy, Monterotondo Scalo, Rome, Italy; 6 Institute of Translational Pharmacology, National Research Council of Italy, Rome, Italy; Imperial College London, United Kingdom

## Abstract

Global gene expression profiling was performed using RNA from human embryonic neural stem cells (hENSC), and adult human olfactory bulb-derived neural stem cells (OBNSCs), to define a gene expression pattern and signaling pathways that are specific for each cell lineage. We have demonstrated large differences in the gene expression profile of human embryonic NSC, and adult human OBNSCs, but less variability between parallel cultures. Transcripts of genes involved in neural tube development and patterning (ALDH1A2, FOXA2), progenitor marker genes (LMX1a, ALDH1A1, SOX10), proliferation of neural progenitors (WNT1 and WNT3a), neuroplastin (NPTN), POU3F1 (OCT6), neuroligin (NLGN4X), MEIS2, and NPAS1 were up-regulated in both cell populations. By Gene Ontology, 325 out of 3875 investigated gene sets were scientifically different. 41 out of the 307 investigated Cellular Component (CC) categories, 45 out of the 620 investigated Molecular Function (MF) categories, and 239 out of the 2948 investigated Biological Process (BP) categories were significant. KEGG Pathway Class Comparison had revealed that 75 out of 171 investigated gene sets passed the 0.005 significance threshold. Levels of gene expression were explored in three signaling pathways, Notch, Wnt, and mTOR that are known to be involved in NS cell fates determination. The transcriptional signature also deciphers the role of genes involved in epigenetic modifications. SWI/SNF DNA chromatin remodeling complex family, including SMARCC1 and SMARCE1, were found specifically up-regulated in our OBNSC but not in hENSC. Differences in gene expression profile of transcripts controlling epigenetic modifications, and signaling pathways might indicate differences in the therapeutic potential of our examined two cell populations in relation to in cell survival, proliferation, migration, and differentiation following engraftments in different CNS insults.

## Introduction

Multipotent neural stem cells (NSC) that are capable of self-renewal and generate all three cell types of the central nervous system (neurons, oligodendrocytes, and astrocytes) are presently the research hotspot in neuroscience. In the adult mammalian brain, the subependymal layer of the lateral ventricles houses neural stem cells giving rise to young neurons migrating towards the olfactory bulb [1]. NSCs can be isolated from human fetal brain tissue [1], [2] as well as from several regions of the adult human brain such as olfactory bulb (OB), cortex, hippocampus, or subventricular zone (SVZ) of the lateral ventricles [3]–[10]. In humans, a lateral ventricular extension of the migratory stream to the OB has recently been demonstrated and neural stem/progenitor cells (NS/PCs) have successfully been isolated from the OB, which therefore represents an accessible source of neural precursors [11]–[13]. Due to their ability to self-renew and to differentiate towards the neuronal phenoype, human adult olfactory bulb neural stem cells (OBNSCs) provide an attractive tool for transplantation-based therapy of neurodegenerative diseases that avoids the ethical issues raised by the use of human embryos [14]–[23]. Even though adult OBNSC are lineage restricted, which means that they can differentiate only into cells of their tissue origin, there is a growing body of evidence that these stem cells can break the barriers of germ layer commitment [23].

Although there is a great interest and potential of adult human olfactory bulb NSC (OBNSC) in cell replacement therapy, there is lack of data about their gene expression profiling, and molecular pathways that govern their multipotency, proliferation, migration, and signaling mechanisms. A better understanding of the molecular basis of the aforementioned processes would facilitate development of new therapeutic strategies for different neurodegenerative and traumatic diseases of the CNS.

Previous genomic profiling of human embryonic NSC found expression of various genes related to stemness, multipotency, and neuroectodermal cell fate [24], [25]. Cai and colleagues found expression of core neural stem cell markers, such as Nestin, Prominin1, SOX1, and SOX2 [24]. Our group had previously found that the set of genes expressed more highly in human embryonic NSCs is enriched in molecules known or predicted to be involved in M phase of mitotic cell cycle [26]. To our knowledge, comparing the transcriptional profile of adult human OB-NSC with other NSCs from embryonic, fetal, and adult tissue is still lacking. Moreover, clarifying differences in expression profile of genes known to control epigenetic alterations between the two cell classes is crucial to provide insight about their future therapeutic potential following engraftment. In this study, we focus on comparing the genomic profiles and signal pathway analysis of human adult olfactory bulb and embryonic NSCs using oligonucleotide microarrays and immunocytochemistry to provide a). knowledge of the gene expression profiles and alternative signaling pathways of adult human OB-NSC, and whether adult human OB-NSCs are identical to the embryonic ones; b). to determine how the gene expression patterns of a adult OB-NSCs change and whether its potency becomes narrowed in comparison to embryonic ones, and c. to clarify possible epigenetic alteration between the two cell classes. The gene expression profiling of adult human OB-NSCs was also compared with different data sets from other stem cell populations: a. a pluripotent stem cell derived from the inner cell mass and hence without organ assignment (an ESC); b. embryonic neural cells isolated and maintained primarily as neurospheres; c. a multipotent stem cell from another organ system (the human mesenchymal stem cells, hMSC); d. adult human neuroprogenitor cells, and e. fetal human nuroprogenitor cells.

## Materials and Methods

### Culturing of Human Embryonic NSCs

Five cell samples were selected for expression profiling as indicated in Table S1. Cryopreserved human embryonic neural stem cells were plated in a 6-well culture plate coated with polyethyleneimine, and incubated at 37°C in a 5% CO2/95% air incubator in serum-free DMEM/F-12 medium (Invitrogen, Carlsbad, CA, USA) supplemented with a mixture of insulin–transferrin–selenium (ITS) (Invitrogen, Carlsbad, CA, USA), 20 ng/ml recombinant human EGF (Invitrogen, Carlsband, CA), 20 ng/ml recombinant human bFGF (Invitrogen, Carlsband, CA), and 10 ng/ml recombinant human LIF (Invitrogen, Carlsband, CA), according to the methods described previously [27] ^.^ The half of the medium was renewed every 4 days. Following incubation for several months, the embryonic NSC in culture continued to proliferate by forming free floating or loosely attached growing spheres. For microarray analysis, nonpassage embryonic NSC spheres were harvested, replated in a noncoated 6-well culture plate, and incubated further for 72 h in the NSC medium without inclusion of 10% fetal bovine serum (FBS) (Invitrogen, Carlsbad, CA, USA).

### Isolation, and Culturing of Human Olfactory Bulb NSCs

The OBs were harvested from adult patients undergoing craniotomy at the Institute of Neurosurgery, Catholic University, Rome (Table S1). Informed consent was obtained according to protocols approved by the Ethical Committee of the Catholic University. Immediately after removal, the OBs were dissociated in Papain 0.1% (Sigma-Aldrich, St. Louis, MO) for 30 minutes at 37°C. Dissociated cells were cultured in the presence of human recombinant EGF (20 ng/ml; PeproTech, Rocky Hill, NJ), human recombinant bFGF [10 ng/ml; PeproTech), and LIF (20 ng/ml; Immunological Sciences, Rome, Italy) in DMEM/F12 (1∶1) serum-free medium (Invitrogen, Carlsband, CA) containing L glutamine 2 mM, glucose 0.6%, putrescine 9.6 ug/ml, progesterone 0.025 mg/ml, sodium selenite 5.2 ng/ml, insulin 0.025 mg/ml, apo-transferrin sodium salt 0.1 mg/ml, sodium bicarbonate 3 mM, Hepes 5 mM, BSA 4 mg/ml, heparin 4 ug/ml. Primary neurospheres were dissociated with Accutase (Invitrogen) for 4 minutes at 37°C, serially diluted and plated one cell per mini-well onto 96-well plates. Mini-wells containing one single cell were marked after microscopic confirmation and assessed for secondary neurosphere generation after one week. Secondary neurospheres were subsequently dissociated, plated at the density of 10^3^ cells/cm^2^ in serum-free medium containing EGF and bFGF, and passaged up to P30. Between P7 and P10, parallel cultures were established in which cells were grown as adherent monolayers in medium containing EGF and bFGF supplemented with 5% fetal calf serum (Hyclone, Logan, UT). Cells were counted with hemacytometer every 48 hours. Differentiation assays were performed by 14 days after plating on Matrigel coated glass coverslips in the absence of EGF and bFGF and in the presence of 1% fetal calf serum (Hyclone) supplemented with 3′–5′-cyclic adenosine monophosphate (cAMP) 50 mM, all-trans retinoic acid 5 mM (Sigma Aldrich), and triiodothyronine (T3) 30 nM (Sigma Aldrich) [28].

### Illumina bead chip hybridizations and analysis of expression data

Total cellular RNA was isolated from passage three embryonic NSC (n = 5), and passage twenty two adult human OB-NSCs (n = 6) using the Trizol (Invitrogen). Approximately 400 ng of total RNA from each sample served as the input to generate biotinlabeled cRNA using a linear amplification kit (Ambion, Austin, TX, United States). RNA and biotinylated cRNA concentrations were confirmed with Nanodrop ND-1000 and controlled for quality using a BioRad Experion electrophoresis station. Next, cRNA samples (750 ng) were hybridized onto Illumina SentrixH HumanHT-12 v3 Expression Bead Chips at 58°C overnight [19 h]. Chips were scanned with the Illumina Bead Array Reader (Factor  =  1, PMT =  520, Filter  =  100%), and the numerical results were extracted with GenomeStudio using the Gene Expression Module v.1.0.6. Raw data were background-subtracted and normalized using the quantile normalization method (lumi software package) [28], [29]. Normalized data were filtered for genes with significant expression levels compared to negative control beads. Selection for differentially expressed genes was performed on the basis of arbitrary thresholds for fold changes plus statistical significance according to the Illumina t-test error model (limma software) [30]. The mRNA array data in MIAME compliant and has been submitted to the NCBI Gene Expression Omnibus (GEO) database (Accession: GSE35390).

### Data Analysis

#### Gene Ontology Analysis

We identified gene ontology [GO] groups of genes whose expression was differentially regulated among the classes. By analyzing GO groups, rather than individual genes, we were able to reduce the number of tests conducted, and to enable findings among biologically related genes to reinforce each other. This analysis is different than annotating a gene list using GO categories. For each GO group we computed the number n of genes represented on the microarray in that group, and the statistical significance pi value for each gene i in the group. These p values reflect differential expression among classes and were computed based on random variance t-tests or F-tests [31]. For a GO group, two statistics are computed that summarize the p values for genes in the group; the Fisher (LS) statistic and the Kolmogorov-Smirnov (KS) statistic as described in [32]. Samples of n genes are randomly selected from genes represented on the array and the summary statistic computed for those random samples. The significance level associated with the GO category is the proportion of the random samples giving as large a value of the summary statistic as in the actual n genes of the GO category. For each GO category, two significance levels are computed, corresponding to the two summary statistics. We considered a GO category significantly differentially regulated if either significance level was less than 0.01. We considered all GO categories with between 5 and 100 genes represented on the array. Some of the categories were overlapping.

The evaluation of which Gene Ontology classes are differentially expressed between pre and post treatment samples was performed using a functional class scoring analysis [33]. For each gene in a GO class, the p value for comparing pre versus post treatment samples was computed. The set of p values for a class was summarized by two summary statistics: (i) The LS summary is the average log p values for the genes in that class and (ii) the KS summary is the Kolmogorov-Smirnov statistic computed on the p values for the genes in that class. The statistical significance of the GO class containing n genes represented on the array was evaluated by computing the empirical distribution of these summary statistics in random samples of n genes. Functional class scoring is a more powerful method of identifying differentially expressed gene classes than the more common over-representation analysis or annotation of gene lists based on individually analyzed genes. The functional class scoring analysis for Gene Ontology classes was performed using BRB-ArrayTools.

All significantly expressed transcripts (P≤0.05, FC≥1.5) were clustered using a hierarchical clustering method. The determination of the correct number of clusters was based on measuring the similarity of each gene to its own cluster compared to the similarity of the gene to genes in other clusters, which was measured using the average of the intracluster and intercluster distances. MATLAB software (v. 7.3) was used for clustering and correlation. Expander software (v. 5.07) [34] was used for the hierarchical clustering of transcripts overexpressed in each stage separately and cell cycle associated transcripts. Using the average linkage method, transcripts were clustered, and the expression matrix was visualized with a dendrogram. The STRING database (version 8.1) [35] was used to construct a regulatory network of differentially expressed transcripts. The visualization of networks was performed using Cytoscape (version 2.6.3) [36]. We used BiNGO (a Cytoscape plugin) [37] to find statistically over- or underrepresented Gene Onthology (GO) categories in the biological data as a tool to enrich the analysis of the transcriptome dataset. Enrichment was determined in reference to all human Entrez GeneIDs that were annotated in the Biological Process branch [14,394 genes total]. P-values were derived from a hypergeometric test followed by the Benjamini and Hochberg false discovery rate [38]. A P-value cutoff of 0.01 was used to identify significantly enriched categories. Pathway analyses were assigned with the ClueGO (v. 1.2) plugin to all of the genes using the KEGG database. A two sided hypergeometric test was used as statistical test for the probability of each gene falling into a pathway.

#### Functional Annotation and Molecular Network Analysis

Functional annotation of significant genes identified by microarray analysis was searched by the web-accessible program named Database for Annotation, Visualization and Integrated Discovery (DAVID) version 2009, National Institute of Allergy and Infectious Diseases (NIAID), National Institutes of Health (NIH) (david.abcc.ncifcrf.gov) [39], [40]. DAVID covers more than 40 annotation categories, including Gene Ontology (GO) terms, protein–protein interactions, protein functional domains, disease associations, biological pathways, sequence general features, homologies, gene functional summaries, and tissue expressions. By importing the list of the National Center for Biotechnology Information (NCBI) Entrez Gene IDs, this program creates the functional annotation chart, an annotation-term-focused view that lists annotation terms and their associated genes under study. To avoid excessive count of duplicated genes, the Fisher’s exact test is calculated based on corresponding DAVID gene IDs by which all redundancies in original IDs are removed. Gene ontology (GO) and KEGG molecular pathway analysis was performed to identify possible enrichment of genes with specific biological themes using both the data set as a whole and then in the individual K-means clusters. DAVID calculates a modified Fishers Exact p-value to demonstrate GO or molecular pathway enrichment, where p-values less than 0.05 after Benjamini multiple test correction are considered to be strongly enriched in the annotation category.

#### Strategy for selecting genes potentially involved in motility, stemness, proliferation and migration

From the results of microarray analysis, we sought to identify genes which were of biological interest, i.e., which were directly relevant to the basic mechanisms of NSC. Genomic databases (Gene) and published work from the scientific literature (PubMed) were used to select genes involved in: (1) motility and migration in embryogenesis, with particular reference to neural stem cells; (2) stemness and pluripotent capacity of NSCs; (3) cell proliferation and/or migration in general.

#### Immunohistochemical validation of selected genes

For immunocytochemistry, NPSs attached on poly-L-lysine-coated cover glasses were fixed with 4% PFA in 0.1 M phosphate buffer, pH 7.4 at room temperature (RT) for 5 min, followed by incubation with phosphate-buffered saline (PBS) containing 0.5% Triton X-100 at RT for 3 min. After blocking non-specific staining by PBS containing 10% NGS, the cells were incubated in primary antibodies. We used antibodies against nestin (Chemicon, Temecula, CA), GFAP (Dako, Glostrup, Denmark), β-tubulin III (Chemicon), neurofilament RT-97 (Developmental Studies Hybridoma Bank, Iowa City, IA), MAP2 (Chemicon), NG2 (Chemicon), O4 (Chemicon), and hTERT (Novocastra Laboratories). Then, they were incubated at RT for 30 min with a mixture of FITIC-conjugated anti-rabbit IgG (Invitrogen) and TRITIC-conjugated anti-mouse IgG (Invitrogen). After several washes, they were examined on the Olympus BX51 universal microscope. Immunostaining of NS/PCs was performed as described^14^.

## Results and Discussion

### Embryonic Human NSCs in Culture

**Figure 1:**
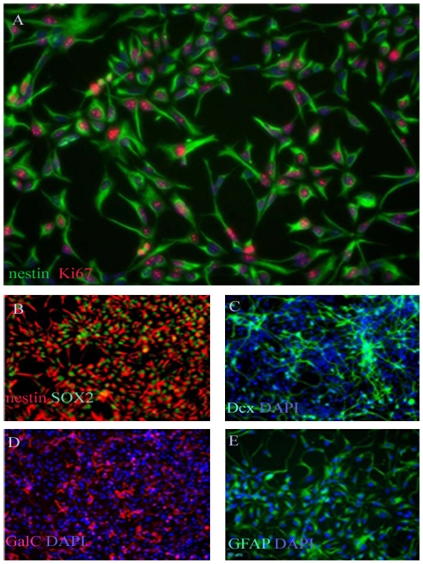
Fluorescence image (20X) of GIBCOR hNSCs at passage 3 that have been cultured in StemProR NSC SFM and stained for the NSC phenotype markers nestin (green) and the proliferation marker Ki67 (red, a). Cell nuclei were counterstained with DAPI (blue,a). Approximately 90% of the cells stain positive for the undifferentiated NSC marker nestin and the proliferation marker Ki67. Lack of Oct4 staining indicates that there are no remnant hESCs in the culture (data not shown) (Invitrogen, Manual part no. A11592, MAN0001758). Fluorescence images (20X) of GIBCOR hNSCs that have been cultured in StemProR NSC SFM for three passages, and then allowed to differentiate into neurons, oligodendrocytes, or astrocytes. Upon directed differentiation, cells start to lose the undifferentiated NSC marker, nestin, but stain positive for the differentiated cell type markers Dcx, GalC, and GFAP. Cells were stained for the undifferentiated NSC markers nestin (red, b) and SOX2 (green, c) prior to directed differentiation. Cell were then differentiated into neurons and glial cells, and respectively stained for the neuronal marker Dcx (green, c), for the oligodendrocyte marker GalC (red, d), or for the astrocyte marker, GFAP (green, e). The nuclei were counterstained with DAPI (blue) in panels B–D (Invitrogen, Manual part no. A11592, MAN0001758).

Five specimens of human embryonic NSCs (hENSC) were used in the current studies (Table S1). A single cell suspension of hENSC were cultured in serum-free DMEM/F12 medium supplemented with EGF and bFGF. The five specimens gave rise to proliferating neurospheres, first appeared within 72 h of primary culture and increased their numbers and diameters quickly during 7 days after the onset of the culture. The neurospheres were splitted into single cells using accatase. Approximately 90% of the cells stained positive for the undifferentiated NSC marker nestin, SOX2 and the proliferation marker Ki67 (Figure 1A, B). Lack of Oct4 staining indicates that there are no remnant hESCs in the culture (data not shown). The multipotency of these human embryonic cell-derived NSC was confirmed by differentiation assay in vitro. The differentiation capacity of ENSC in differentiating conditions was revealed by examining the types of molecular markers expressed by neurons and glial cells. These cells could differentiate into, Dcx-positive immature neurons (Figure 1C), GalC positive oligodendrocyte (Figure 1D), and GFAP positive astrocyte (Figure 1E). The hENSCs could be passed at least for ten generations by mechanical dissociation and their stemness and multipotency could be maintained in serum-free medium supplemented with growth factors for at least one month.

### Adult Human OB-NSCs in Culture

**Figure 2:**
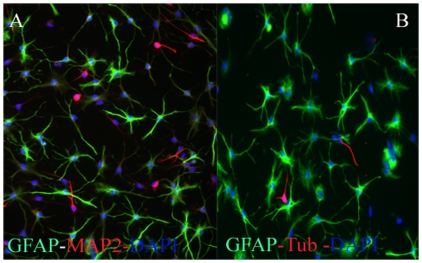
Differentiation potential of short-term proliferated human OBNS cells. Fluorescent Phase contrast images of passage 9 human OBNS immunostained for the GFAP astrocytes marker, MAP2 immature neuronal marker, and β-Tubulin mature neuronal marker. Scale bar, 100 µm. The nuclei were stained blue with DAPI. The plot shows the percentage of positive GFAP astrocytes, MAP2 immature, and β-Tubulin mature neurons, generated by each cell type.

**Figure 3:**
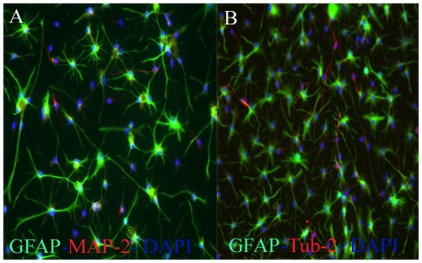
Differentiation potential of long-term proliferated human OBNS cells. Fluorescent Phase contrast images of 20 human OBNS immunostained for the GFAP astrocytes marker, MAP2 immature neuronal marker, and β-Tubulin mature neuronal marker. Scale bar, 100 µm. The nuclei were stained blue with DAPI. The plot shows the percentage of positive GFAP astrocytes, MAP2 immature, and β-Tubulin mature neurons, generated by each cell type.

Dissociated adult human OB specimens were cultured in serum-free medium supplemented with the mitogens EGF and bFGF. Under these conditions, the OB cells generated primary neurospheres with latencies that ranged from 6 to 8 weeks. Adult Human OB-NSCs were capable of proliferating for several months by forming free floating or loosely attached growing spheres, when incubated in the NSC medium under the serum-free culture conditions. When adult human OB-NSC spheres were split into single cells with accutase and incubated in the NSC medium supplemented with 10% FBS, they rapidly attached on the plastic surface, and started to differentiate into the different neuronal and glial lineage. To study the effects of culture time on the differentiation potential of adult human OB-NSC, we studied their differentiation potential during short (passage 9), and long (passage 20)-term proliferation. Short term proliferated human ON-NSC exhibited an intense immunoreactivity for GFAP astrocytes marker (75%), MAP2 immature neuronal marker (17.5%), and β-Tubulin mature neuronal marker (5%) (Fig. 2 A,B). In comparison, the differentiation potential of long-term differentiated ones were 75±4.9%, 12.5±2.4%, and 17.5±5.2% for GFAP, MAP2, and β-Tubulin immunoreactivity respectively (Fig. 3 A, B). None of the cells expressed the oligodendrocyte marker O4 in short and long-term proliferation culture conditions (data not shown). These results suggest that during short and long-term culture conditions, the human OB-NSC were differentiated into astrocytes (75%), immature (12.5–17%) and mature (5–17.5%) neurons. Moreover, the neurogenic differentiation potential of human OB-NSC increased during long-term culture conditions.

### Human OBNSC Show Stable Gene Expression Over an Extended Period of Time

In order to establish basal gene expression in our human OBNSC, and begin to explore the differences between them and human ENSC, the pattern of gene expression in our human OBNSC was compared over time. Adult human OBNSC was grown for 20 weeks in EGF and FGF containing media. Total RNA was harvested at 5 and 25 weeks of culture (P9, and P 25), and their gene expression profiles were compared using Illumina microarray. Illumina Microarray Suite 4.1 was used to scan and analyze the relative abundance of each gene. The total gene expression levels in human OBNSC (Table S2) were stable for approximately 25 weeks in the long-term presence of EGF and FGF. The gene array set contains 47232 expressed genes and expressed sequence tags (ESTs). Only 94 genes were differentially expressed between our two examined cellular classes, OBNSC Vs. hENSC (Table S3) which were nearly equal to the number of false positive signals. These finding might indicate that there was no significant differences in the gene expression profiles of our examined cell population over prolonged period of culture, and that OBNSCs were genetically stable during our examined time scale.

Next, we examined genes that are important in the development of the nervous system. As expected, nestin, notch, and the EGF receptor were up-regulated in both cell populations. Several genes identified as stem-cell specific including Slit2, and insulin-like growth factor binding protein 2 was also present in OBNSC. Members of bone morphogenetic protein (BMP) family which is involved in epidermal induction [41] and glial fate [42], genes for growth factors such as brain-derived neurotrophic factor (BDNF), glial derived neurotrophic factor (GDNF), FGF-2 and its receptor FGFR1, FGF-8, and about 22 members of wnt family are identified in our OBNSC. The full list of up and down regulated genes across the five human OBNSC replicates can be found in Table S4.

### Transcriptome Analysis of Human Embryonic NSC versus Human Adult OB-NSC

**Figure 4:**
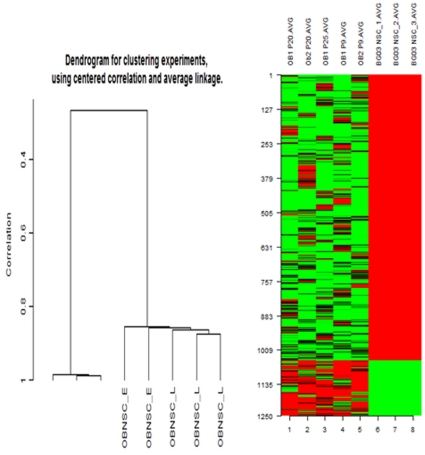
Hierarchical clustering and interclass correlations between hESCs and OBNSCs. A. Hierarchical clustering of 1252 differentially expressed genes was performed using the mean signal intensity for each replicate. Biological replicates of hESCs and OBNSCs were compared and showed high intraclass correlations compared with interclass correlations. Two distinct clusters were distinguishable based on the expression patterns of the different cell types. The differentially expressed transcripts were clustered into two expression groups, including 203 genes that were up regulated in OBNSC compared to 1049 genes that were up regulated in hENSC. B. Expression patterns of representative genes from different expression clusters. Transcripts that are highly up-regulated in hENSC (red) compared with the OBNSC (green).

Hierarchical clustering dendrogram of relative gene expression in different populations of OBNSC and human ENSC was generated using the GeneSpring GX Software. hENSCs clustered together and are distinguished from human OBNSC cells. There was no correlation between hENSC and adult human OBNS cells samples. But there was a strong correlation between the arrays from the same samples. Unsupervised hierarchical clustering based on overall gene expression profiles reveals two distinct clusters corresponding to the two cell groups. This suggests that the samples from the two cell types (hENSC vs OBNSC cells) do indeed represent two statistically distinct populations suitable for valid comparison (Fig. 4 A, B)**.** An analysis of transcriptome dynamics between hENSC and OBNSC revealed that 1252 transcripts were modulated between the two cell populations (Table S5). While most 83% (1049) of the modulated genes were up-regulated in hENSC, only 17% (203) of modulated genes were up-regulated in OBNSC.

### Up-regulated Genes in Both Human ENSC and OBNSC

Transcripts of genes involved in neural tube development and patterning (ALDH1A2, FOXA2), progenitor marker genes (LMX1a, ALDH1A1, SOX10), proliferation of neural progenitors (WNT1 and WNT3a), neuroplastin (NPTN), POU3F1 (OCT6), neuroligin (NLGN4X), MEIS2, and NPAS1 were up-regulated in both OBNSC and hENSC. Neuroplastin NPTN is a glycoprotein that belongs to the immunoglobulin superfamily of cell adhesion molecules (CAMs). This gene is also involved in the long-term potentiation of hippocampal excitatory synapses through the activation of p38MAPK [43]. Recently, it was demonstrated that neuroplastin binds to and activates fibroblast growth factor receptor 1 (FGFR1) [44], and it may have a function in FGF signaling in OBNSC and hESCs. Some antagonists of FGF signaling, such as SPRY1, were also overrepresented in OBNSC and hESCs. SPRY1 is involved in cortical neuron pattern formation and inhibits caudal cell fates [45]; its role in human embryonic stem cells (hESCs) with a high concentration of FGF is not clear and may be important for the fine tuning of FGF signaling in ESCs. POU3F1 is a member of the pou domain family of proteins and is involved in neural ectoderm formation; its expression is down regulated upon ESC differentiation and increases again during brain development [46]. NLGN4X is a putative neuronal cell surface protein involved in cell-cell-interactions and may be involved in the formation and remodeling of central nervous system synapses [47]. During the early development of neural cells, the MEIS2 homeobox genes can positively control PAX6 transcription and induce hESCs toward neutralization [48]. The protein encoded by the NPAS1 gene is a member of the basic helix-loop-helix (bHLH)-PAS family of transcription factors and is specifically expressed in neural tissue. NPAS1 in mice modulates the transcription of erythropoietin by binding to its enhancer region in vivo; thus, it indirectly controls oxygen responsive elements during late embryogenesis and central nervous system development [49].

Other genes that were up-regulated in our OBNSC and hENSCs are CYP26A1, CPZ, and FGF8. CYP26A1 plays a key role in retinoic acid (RA) metabolism, highlighting the importance of the retinoic acid (RA) metabolic pathway in the neural initiation stage [50]. CPZ, modulates the WNT signaling pathway by cleaving some undefined protein or by binding to the WNT molecule [51]. FGF8 a paracrine factor that is downregulated as differentiation progresses, and has a function during dopaminergic neuron specification and proliferation [52]; it works cooperatively with SHH in the specification of midbrain neurons. Shh protein can induce Foxa2 and ventralize neural progenitors and, in a positive regulatory loop, FOXA2 can induce endogenous SHH and inhibit NKX2.2 [53] and also the serotonergic phenotype. Endogenous transcription of FGF8 resulting from RA exposure [54] can induce WNT1 expression that cooperatively with FGF8 can induce neural progenitors to differentiate into TH-producing cells. Matrix associated genes, including MMP1, THBS1, and ITGB1BP3, were also among our OBNSC, and hESC enriched genes, suggesting that expression of these genes provides an environment conducive to the proliferation of stem cells. As expected, major markers of pluripotent hES cells, including NANOG, OCT4 (POU5F1), REX1 (ZFP42), FGF4, FOXD3, CLDN6, GDF3, DNMT3A, and CD2, were up-regulated in both NPCs and MPCs. Both cell types overexpressed other genes commonly associated with a neural stem/progenitor cell fate: Jagged 1 (JAG1) [55], SOX2 [56], SOX4 [57], Nestin (NES) [58], the oligodendrocyte lineage transcription factor two [OLIG2] [59], the G protein-coupled receptor 56 (GPR56) [60], the vascular endothelial growth factor (VEGF), and the stem cell marker Musashi1 (MSl1) [60]. Genes associated with cell cycle progression, such as a disintegrin and metalloproteinase domain nine (ADAM9), HAT1-, protein kinase-, DNA-activated, catalytic polypeptide (PRKDC), or RNA binding motif protein 3 (RBM3) were up-regulated in both cell types.

### Genes Specific for Adult OB-NSC

The up-regulation of 203 genes and the down-regulation of 1049 in our OBNSC in comparison to hENSC might point to peculiar alternative metabolic pathways for each cell class. We found that a number of genes specific for neurons were transcribed only on reduced levels: the 25-kDa synaptosome-associated protein (SNAP25), neurogranin (NRGN), and others. When looking for genes specifically expressed in our OBNSCs, a large number of such genes can be retrieved. The low-affinity nerve growth factor receptor precursor (p75, NGFR), Nestin (NES), and chondroitin sulfate proteoglycan four (CSPG4) were specifically highly expressed in the OBNSC (Table S4). The gene CSPG4 encodes for the epitope NG2, which is reported to be specifically expressed on oligodendrocyte precursor cells. Genes associated with cell cycle progression, such as a disintegrin and metalloproteinase ndomain nine (ADAM9), HAT1-, protein kinase-, DNA-activated, catalytic polypeptide (PRKDC), or RNA binding motif protein 3 (RBM3) were up regulated in OBNSC but not in hENSC.

The expression pattern of OBNSs lacked the mRNA for myelin components of mature oligodendrocytes: myelin basic protein (MBP), proteolipid protein one (PLP1), and others. The neurofilament heavy chain (NEFH) or the GABAreceptor, alpha 1 (GABRA1) were upregulated (Table S4). A number of SOX- and homeobox genes were highly expressed: SOX4, SOX11, SOX12, and the LIM homeobox gene two (LHX2), or the distal-less homeobox gene 2 (DLX2). In addition, we could detect an up-regulation of prominin one (PROM1 or CD133) in OBNSC. Cell proliferation-like topoisomerase II-(TOP2A) genes were uniquely overexpressed in OBNSC.

The α-receptor of platelet derived growth factor (PDGFRa) was expressed in OBNSC only. As reported earlier, even fetal hNPCs showed a high expression of PDGFRα absent both [61]. As reported for PDGF, a factor influencing proliferation, differentiation and migration of cells [62], this may indicate a pivotal role in stem cell recruitment and hence the expression of its receptor on stems cells in general. PDGFRα-signaling occurs early in the adult stem cell lineage and may help regulate the balance between oligodendrocyte and neuron production [63].

### Comparative Transcriptome Analysis of Human hENSC and OBNSC

We then compared the complex transcriptomes of hENSCs with adult human OBNSCs. Global comparison between hENSCs and OBNSCs however showed that both NSC types differ in various gene categories, with the largest differences in “macromolecular complex assembly (10 genes), macromolecular complex subunit organization (10 genes), cellular macromolecular complex assembly (6 genes), cellular macromolecular complex subunit organization (6 genes), protein complex assembly (7 genes), protein complex biogenesis (7 genes), ribonucleoprotein (19 genes), cytosolic ribosome (13 genes), ribosomal protein (16 genes), translational elongation (13 genes), protein biosynthesis (13 genes), with an enrichment score of 6.78 (Table S5). Despite these global differences between the transcriptomes of hENSCs and OBNSCs, major similarities could be observed (Table 1, and Table S5): Most of these similarities are based on genes being related to neuronal and neuroendocrine function, signaling cell membrane and extracellular matrix (Table 1, and Table S5): Brain and acute leukemia gene (BAALC), normally expressed in brain by neurons and endocrine cells [64] and highly conserved among mammals [65], glycoprotein 6B (GPM6B), endothelian receptor A (EDNRA), and Stathmin-like 2 (SCGN10) were as well highly expressed in hENSCs and OBNSCs. Genes involved in fat metabolism such as apolipoprotein D were also up-regulated in hENSCs and OBNSCs. The SLC6A15 (solute carrier family 6, member 15) and S100 calcium binding protein B, NeuroD1 and OTX1 were highly expressed in both cell types. However, previous cell biology investigations including differentiation studies of hmNSCs demonstrated all major properties of NSCs including differentiation into functional glia and neurons [66]–[68], while they lost their mesodermal stromal cell characteristics [66]. Noteworthy, we previously found extensive differences of transcriptomes even between various “primary” NSC types including particularly the expression of stem cell markers suggesting divergent paths to maintain the neuroprogenitor cell state [61].

To further confirm our results, we compared the global genomic profiling of our data with several other data sets from adult neuro progenitor cell (aNPCs), fetal neuro progenitor cell (fNPCs), human mesenchymal stem cells (hMSCs), and adult hippocampal tissue (aHypp). Genes that were up-regulated in both human OBNSC and hENSC include various genes related to differentiated neurons and glia, such as MAPT [microtubule associated protein tau] and other tubulin associated genes, synapse formation (SNAP25), and axon guidance (ROBO-SLIT) molecules (Table S3). We have observed up-regulation of stem cell markers such as NES, PROM1, SPRY1, CXCL12, CXCR4. We also observed up-regulation of axon guidance cues and neurotrophins, including ROBO2 and ROBO3 and their receptors SLIT2 and SLIT3, Eph receptors (EFNB3, EPHA3, EPHA5, EPHA8, EPHB1, EPHB3) and their ligands, and semaphorin receptors (SEMA3E, SEMA4F, SEMA5B, SEMA6C). The protachykinin gene (TAC1), tyrosine hydroxylase (TH) and the somatostatin gene (SST) were also among the highly expressed transcripts in OBNSC (Table S4).

We also compared the expression profile of our OBNSC with hMSCs as a multipotent adult stem cell. We found that hMSCs overexpress a number of genes commonly found in connective tissues (decorin (DCN), fibronectin (FN1), and various collagen proteins), similar to peripheral nervous system (PNS) tissues. However, there are various genes expressed by both OBNSCs and hMSCs, mainly extracellular matrix proteins (COL1A1, COL1A2, COL3A1, LOXL2) and genes such as insulin-like growth- factor 3 and 5 (IGFBP3 and IGFBP5) (Table S4). Other extracellular matrix components genes namely laminin-4 (LAMA4), tenascin C (TNC), and integrin-alpha7 (ITGA7) were also up-regulated in OBNSC (Table S4).

### Functional Annotation Clustering of OBNSC and hENSC

Functional annotation of significant genes identified by microarray analysis was searched by the web-accessible program named Database for Annotation, Visualization and Integrated Discovery (DAVID) version 2009. Clustering for the top 200 up-regulated genes (Log2 176-3.1) of human OBNSC using DAVID, we identified 28 annotation clusters. The annotation cluster 1 (Table S6) showed the highest enrichment score of 1.76 and included genes related to macromolecular complex assembly (10 genes), macromolecular complex subunit organization (10 genes), cellular macromolecular complex assembly (6 genes), cellular macromolecular complex subunit organization (6 genes), protein complex assembly (7 genes), protein complex biogenesis (7 genes). Functional Annotation Clustering using DAVID for the top 200 up-regulated genes of human ENSC identified 61 annotation clusters. The annotation cluster 1 included genes related to ribonucleoprotein (19 genes), cytosolic ribosome (13 genes), ribosomal protein (16 genes), translational elongation (13 genes), protein biosynthesis (13 genes), with an enrichment score of 6.78 (Table S7).

### GO Class Comparison

The categorization of the genes by gene ontology terms were similar in both NPC populations (Fig. S2), whereas the genes themselves overlapped only occasionally [refer to supplemental.

**Figure 5:**
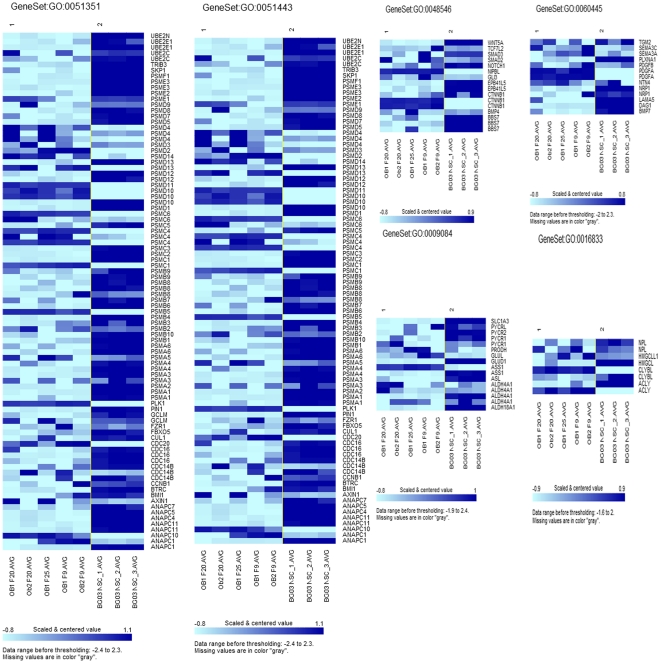
Expression patterns of representative genes from different GO clusters of OBNSC (class 1) and human ENSC (Class 2).

By Gene Ontology: 325 (Figure 5) out of 3875 investigated gene sets passed the 0.005 significance threshold. LS/KS permutation test found 125 significant gene sets. Efron-Tibshirani’s maxmean test found 240 significant gene sets between adult OBNSC, and hENSC (under 200 permutations). 41 out of the 307 investigated Cellular Component (CC) categories were significant. 45 out of the 620 investigated Molecular Function (MF) categories were significant. 239 out of the 2948 investigated Biological Process (BP) categories were significant [Table S8].

### KEGG Pathway Class Comparison

75 out of 171 investigated gene sets passed the 0.005 significance threshold. LS/KS permutation test found 72 significant gene sets between adult OBNSC and hENSC. Efron-Tibshirani’s maxmean test found 17 significant gene sets (under 200 permutations) (Table S9).

### Epigenetic Modification Signatures

The transcriptional signature also deciphers the role of another type of developmental regulation that concerns genes involved in epigenetic modifications. Among genes that are differentially expressed during neural or mesoderm differentiation, genes encoding helicases that function to open chromatin to enhance transcription in the SWI/SNF DNA chromatin remodeling complex family, including SMARCC1 and SMARCE1, were found specifically up-regulated in our OBNSC but not in hENSC and may interact with proteins encoded by other specific genes such as ARID2 and ARID1B [69], [70]. These proteins may play a role in enhancing differentiation by coupling gene repression with global and local changes in chromatin structure [71]. Dnmt3b is found to be up-regulated in our OBNSC but not in hENSC (Table S4). Dnmt3b are expressed highly in the developing mouse embryo and are responsible for global *de* *novo* methylation after implantation [72], consistent with the finding that Dnmt3a can carry out *de novo* methylation in transgenic flies [73]. Dnmt3L, a protein that by itself has no DNMT activity, colocalizes with Dnmt3a and Dnmt3b and is essential for establishing methylation imprints in the female germ line [74], [75]. Last, the deletion of Dnmt2, a member of the DNMT family that lacks biochemically detectable methyltransferase activity, has no obvious phenotype in mice [76]; however, this gene is conserved in Drosophila, highly expressed during oogenesis and may be responsible for the small amount of non-CpG methylation seen in the fly embryo [77], [78]. In general, the known developmental effects of DNA methylation on gene expression involve long-term silencing of gene expression. The attractive idea that genes are transcriptionally activated by removing DNA methylation has lacked strong experimental support until recently. Many correlations between expression and loss of DNA methylation have been reported, and the methylation of some reporters has been shown to inhibit expression. A notable recent example is the human maspin gene, the promoter of which is unmethylated in expressing cells but methylated when silent [79]. Because aging is thought to be one of the most important risk factors for cancer, an age-related predisposition to the hypermethylation of CpG islands that can silence tumor suppressor genes [80] 129 may be one of the factors that could increase the risk of developing of malignancies in some individuals [81], [82], [83].

### Alternative Signaling Pathways Controlling Cell Fate Decisions

Levels of gene expression were explored in 3 signaling pathways, Notch, Wnt, and mTOR signaling pathway that are known to be involved in NS cell fates determination but required different partners depending of lineage-specific differentiation.

#### Notch signaling pathway

About 25 genes encoding for ligands of Notch, such as PSEN2, NUMB, NOTCH4, NOTCH3, NOTCH2, NOTCH1, NCOR2, LFNG, KAT2A, JAG1, HES5, HDAC2, HDACI, EP300, DVL3, DVL2, DTX3, DTX2, DLL3, DLL1, CTR1, APH1A AND ADAM17, were found to be up-regulated in the hENSC but not in OBNSc (Figure S1, S2, Table S9]. Regarding the receptors in this pathway, NOTCH1, NOTCH2, NOTCH3, and NOTCH4 were specifically up-regulated in hENSC, and were found down-regulated in OBNSC. Genes implicated in the modulation of the activity of NOTCH signaling that were specifically up-regulated in hENSCs included LFNG (encoded a fucose-specific glycosyltransferase), ADAM17 (encoded a metallopeptidase involved in the proteolytic release of Notch intracellular domain from the Notch1 receptor), and PSEN1 (presenilins-1) involved in the cleavage of the Notch receptor and the regulation of gamma secretase activity. NUMB encoding for an inhibitor of the Notch pathway and playing a role in the determination of cell fates during development was specifically up-regulated in hENSCs. At least, specific transcriptional factors HES1, downtream targets of Notch signaling, were found specifically up-regulated in OBNSCS but not in hENSC, whereas HES5 was up-regulated in hENSC only. Inhibition of NOTCH can disrupt the maintenance of stem cell characteristics of NPCs [84], [85], by suppressing the HES1 and HES5 genes, which negatively control the expression of the proneural genes MASH1 and NGN1 [86], [87], [88]. As shown previously, NOTCH signaling negatively controls neurogenesis in a stepwise process; in the first step, its activation leads to gliogenesis as opposed to neurogenesis, and in the second step, its activation promotes the production of astrocytes and inhibits the production of oligodendrocytes and neural fates [89]. NOTCH signaling in neural system development has multiple functions. Not only can NOTCH switch a neural cell fate decision [90], but NOTCH signaling also plays an important role in the maintenance of neural stem cells [91]. NOTCH molecules are also needed for cell fate determination in hESCs as they differentiate into the three germ layers [92].

#### Wnt signaling pathway

Concerning the 269 genes involved in the Wnt signaling pathway, up-regulation of 94 genes encoding receptors, legends, and other regulators of this canonical pathway was observed in hENSC, whereas only 24 genes were up-regulated in OBNSC (Figure S3, 4, Table S4). Wnt inhibitors, including secreted antagonists such as DKK1 was up-regulated in hENSC, and down regulated in OBNSC. The expression of WNT1 and most WNT receptors was down-regulated in hENSC, although the expression of WNT8A, WNT9A, WNT7A, WNT5B and WNT2 increased in OBNSC (Table S9). Both cell populations had some overlap in the expression patterns of WNT pathway molecules. The DKK1 gene, an inhibitor of WNT signaling, was up-regulated in hENSCs but not in OBNSCs. However, another inhibitor, DKK2, had an increased expression level in OBNSCs but not in hENSC. Other Wnt inhibitor such as SFRP2 was overexpressed specifically in OBNSCs. In addition, genes encoding for Wnt ligands such as WNT2B, reported to be a repressor of the canonical pathway, appeared to be specifically up-regulated in OBNSC, whereas WNT5A, noncanonical ligand, was found to be up-regulated in hENSC, WNT5B was up-regulated in OBNSC. For the Wnt Receptors, notably Frizzled proteins, FZD, FZD2, 3,4,6,9, were found, respectively, up-regulated or not regulated in hENSCs and OBNSCs. These findings may explain how different members of the WNT gene family may control differentiation of different cell types.

Concerning transcriptional regulators involved downstream of Wnt signaling pathways, genes involved in the repression of the b-catenin complex such as SOX transcription factor (SOX3) was down regulated in both cell population. CTNNBIP1, a gene encoding a small soluble inhibitory protein also termed ICAT (inhibitory of beta-catenin and TCF), which prevents the interaction of b-catenin with different binding partners; including LEF1 was specifically up-regulated in OBNSCs (Table S4).

Gene encoding for the transcription repressor TLE4, a member of the Groucho family, was down regulated in our both cell populations. The expression of genes known to be controlled directly downstream of the canonical b-catenin pathways, some genes, such as DCT was down regulated in both cell types, others genes such as POU3F2, and NRCAM, controlled downstream of the complex containing LEF1, were down regulated in hENSC but not in OBNSCs. Blocking canonical Wnt signaling during post-implantation development increased the number of neural precursors which failed to differentiate to mature neurons, and produced defects of embryonic axis elongation, neurulation and neural tube closure that phenocopy the β-catenin null embryo [93].

#### mTOR signaling pathway

Of the 95 transcripts associated with mTOR signaling pathway, 42 genes were up-regulated in our hENSC, and only 5 transcripts were up regulated in OBNSC. Whereas VEGFB, ULK1, STRADA, RPS6KB2, RPS6, RPKAA1, PIK3R2, MAPK2, and AKT1 were up-regulated in hENSCs, RPS6KB2, RPS6KA3, MLST8, MAPK1, and EIF4E2 were up-regulated in OBNSCs (Figure S5, S6, Table S9]. In embryonic stem cells (ESCs), mTOR signaling can stabilize OCT4, SOX2 and NANOG expression and can negatively control the induction of endoderm and mesoderm from ESCs. Inhibition of mTOR with rapamycin enhanced the expression of endoderm and mesoderm markers and impaired the pluripotency of hESCs, but this effect was not observed in neural differentiation [94]. mTOR signaling functions in neural induction and is involved in the EGF/FGF2-mediated maintenance of neural stem/progenitor cells. Phosphorylated mTOR has been up-regulated in a PI3K-Akt dependent manner during NPC differentiation induced by insulin [95].

#### Cell cycle pathway

Gene expression analysis of 200 transcripts of cell cycle signaling molecules revealed the up-regulation of 113 transcripts in our hENSC, and only 11 transcripts in OBNSC. CDK2, CDK4, CDK6 and molecules involved in the G2-S phase transition, including CDC2, CDC25C and MAD2L1, have higher expression levels in hENSC compared to OBNSCs. TFDP1, another transcription factor that binds to EF1 and controls the transcription of EF1 target genes [95], is also up-regulated in our hENSCs but not in OBNSCs (Figure S7, S8, Table S9).

In conclusions, we have demonstrated large differences in the gene expression profile of human embryonic NSC, and adult human OBNSCs, but less variability between parallel cultures. Transcripts of genes involved in neural tube development and patterning, progenitor marker genes, proliferation of neural progenitors were up-regulated in both our OBNSC and hENSC. By Gene Ontology, 325 out of 3875 investigated gene sets were scientifically different between both cell populations. KEGG Pathway Class Comparison had revealed that 75 out of 171 investigated gene sets were significantly different between the two cell populations. The transcriptional signature also deciphers the role of genes involved in epigenetic modifications. In conclusion, the differences in gene expression of transcripts controlling epigenetic modifications, and signaling pathways might indicate differences in the therapeutic potential of OBNSCs Vs. hENSCs.

## Supporting Information

Figure S1 **Notch signaling pathway**. About 25 genes encoding for ligands of Notch were found to be up-regulated in the hENSC but not in OBNSc. The network was generated by “find significant pathway” function in GeneSpring GX v11 (p<0.05). Connections were based on known interactions between these proteins within the Reactome database. The biological relationship between two proteins is represented as a line.
(TIF)

Figure S2 **Heat map of the Notch signaling pathway forming genes in OBNSC Vs. hENSC.**
(TIF)

Figure S3 **Wnt signaling pathway**. Up-regulation of 94 genes related to this pathway encoding receptors, legends, and other regulators of this canonical pathway was observed in hENSC, whereas only 24 genes were up-regulated in OBNSC.
(TIF)

Figure S4 **Heat map of the Wnt signaling pathway forming genes in OBNSC Vs. hENSC.**
(TIF)

Figure S5 **mTOR signaling pathway.** Of the 95 transcripts associated with mTOR signaling pathway, 42 genes were up-regulated in our hENSC, and only 5 transcripts were up regulated in OBNSC.
(TIF)

Figure S6 **Heat map of the mTOR signaling pathway forming genes in OBNSC Vs. hENSC.**
(TIF)

Figure S7 **Cell cycle signaling pathway.** Gene expression analysis of 200 transcripts of cell cycle signaling molecules revealed the up-regulation of 113 transcripts in hENSC, and only 11 transcripts in OBNSC.
(TIF)

Figure S8 **Heat map of the cell cycle signaling pathway forming genes in OBNSC Vs. hENSC.**
(TIF)

Table S1 Details of Embryonic and adult NSC samples used in the current study.
(DOC)

Table S2 Normalized microarray data of 47232 Illumina probe sets demonstrating the gene expression profile of adult human olfactory bulb neural stem cells (OBNSCs).
(XLS)

Table S3 Genes which are differentially expressed among adult human OBNSCs (class 1), and human embryonic NSCs (class 2).
(XLS)

Table S4 Normalized microarray data of 47232 Illumina probe sets demonstrating the gene expression profile of adult human olfactory bulb NSCs (OBNSCs), and human embryonic NSCs.
(XLSX)

Table S5 1250 modulated genes between human OBNSCs, and hENSC, 203 were up-regulated in OBNSC, and 1049 were up-regulated in hENSC.
(XLSX)

Table S6 Functional Annotation Clustering using DAVID for 203 unregulated genes of human hENSC. 61 annotation clusters were obtained. The annotation cluster 1 included genes related to ribonucleoprotein (19 genes), cytosolic ribosome (13 genes), ribosomal protein (16 genes), translational elongation (13 genes), protein biosynthesis (13 genes), with an enrichment score of 6.78.
(TIF)

Table S7 Functional Annotation Clustering using DAVID for 203 unregulated genes (Log2≤5–12) of human OBNSC. 28 annotation clusters were obtained. The annotation cluster one included genes related to macromolecular complex assembly (10 genes), macromolecular complex subunit organization (10 genes), cellular macromolecular complex assembly (6 genes), cellular macromolecular complex subunit organization (6 genes), protein complex assembly (7 genes), and protein complex biogenesis (7 genes) with an enrichment score of 27.56.
(TIF)

Table S8 Gene Ontology of differentially expressed transcripts in OBNSCs and hENSC. 25 out of 3875 investigated gene sets passed the 0.005 significance threshold LS/KS permutation test found 125 significant gene sets. Efron-Tibshirani’s maxmean test found 240 significant gene sets (under 200 permutations). By Gene Ontologies: 41 out of the 307 investigated Cellular Component (CC) categories are significant. 45 out of the 620 investigated Molecular Function (MF) categories are significant. 239 out of the 2948 investigated Biological Process (BP) categories are significant.
(HTML)

Table S9 KEGG Pathway of differentially expressed transcripts between OBNSCs and hENSCs. 75 out of 171 investigated gene sets passed the 0.005 significance threshold LS/KS permutation test found 72 significant gene sets. Efron-Tibshirani’s maxmean test found 17 significant gene sets (under 200 permutations).
(HTML)
