# Supplementary figures and images for: Gene Expression Profile of Adult Human Olfactory Bulb and Embryonic Neural Stem Cell Suggests Distinct Signaling Pathways and Epigenetic Control
Source: PLoS One. 2012 Apr 2;7(4):e33542. doi: 10.1371/journal.pone.0033542 (PMC3317670; doi:10.1371/journal.pone.0033542)

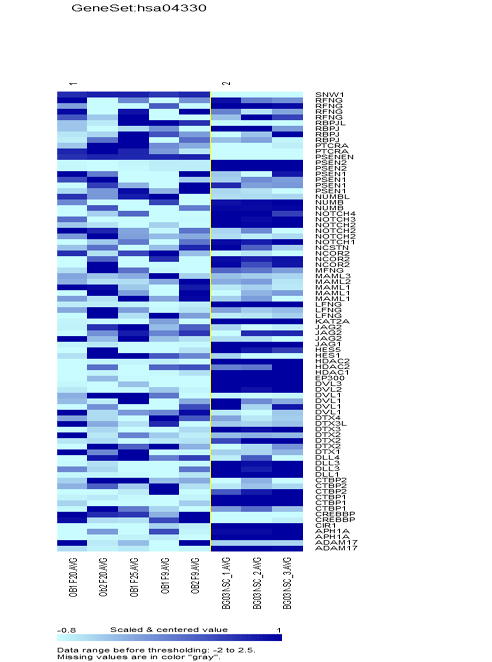

Supplement: Figure S1 — Notch signaling pathway. About 25 genes encoding for ligands of Notch were found to be up-regulated in the hENSC but not in OBNSc. The network was generated by “find significant pathway” function in GeneSpring GX v11 (p<0.05). Connections were based on known interactions between these proteins within the Reactome database. The biological relationship between two proteins is represented as a line. (TIF) [file pone.0033542.s001.tif]

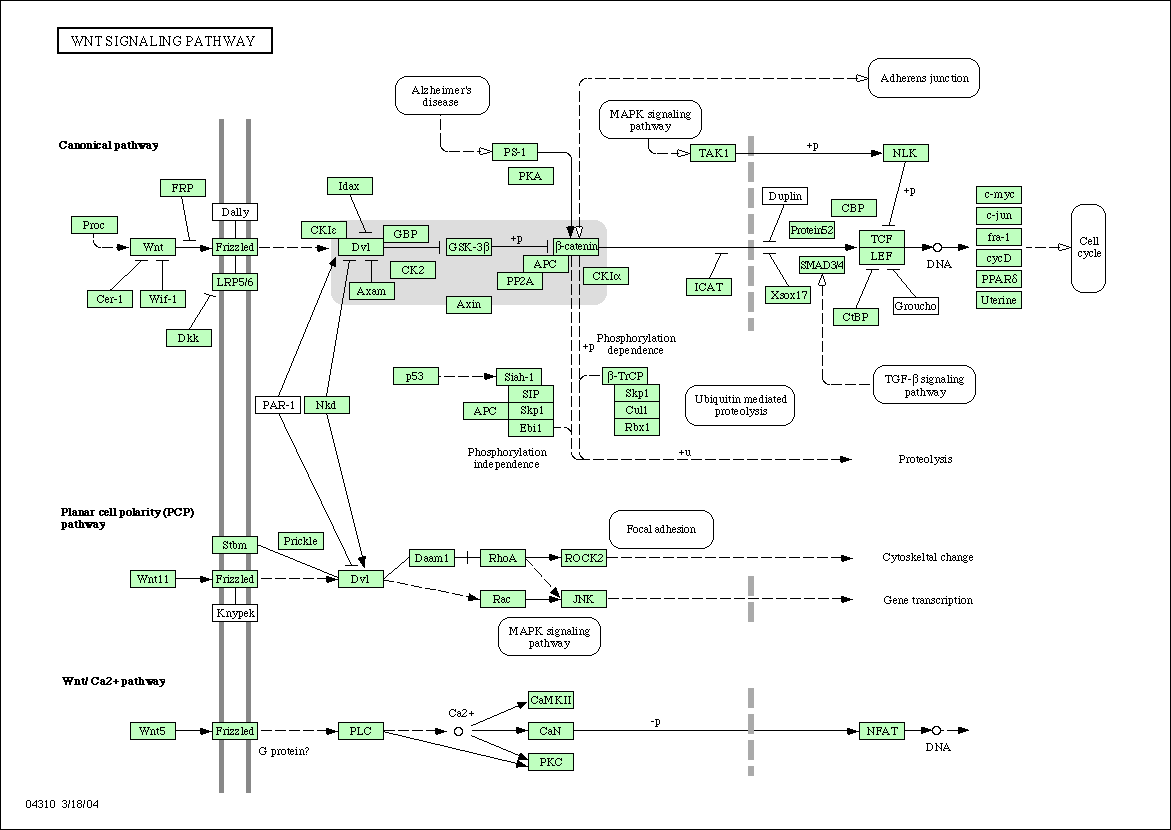

Supplement: Figure S2 — Heat map of the Notch signaling pathway forming genes in OBNSC Vs. hENSC. (TIF) [file pone.0033542.s002.tif]

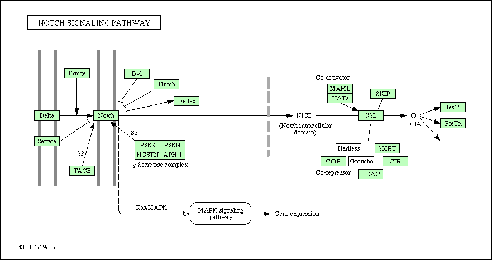

Supplement: Figure S3 — Wnt signaling pathway. Up-regulation of 94 genes related to this pathway encoding receptors, legends, and other regulators of this canonical pathway was observed in hENSC, whereas only 24 genes were up-regulated in OBNSC. (TIF) [file pone.0033542.s003.tif]

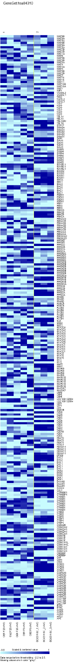

Supplement: Figure S4 — Heat map of the Wnt signaling pathway forming genes in OBNSC Vs. hENSC. (TIF) [file pone.0033542.s004.tif]

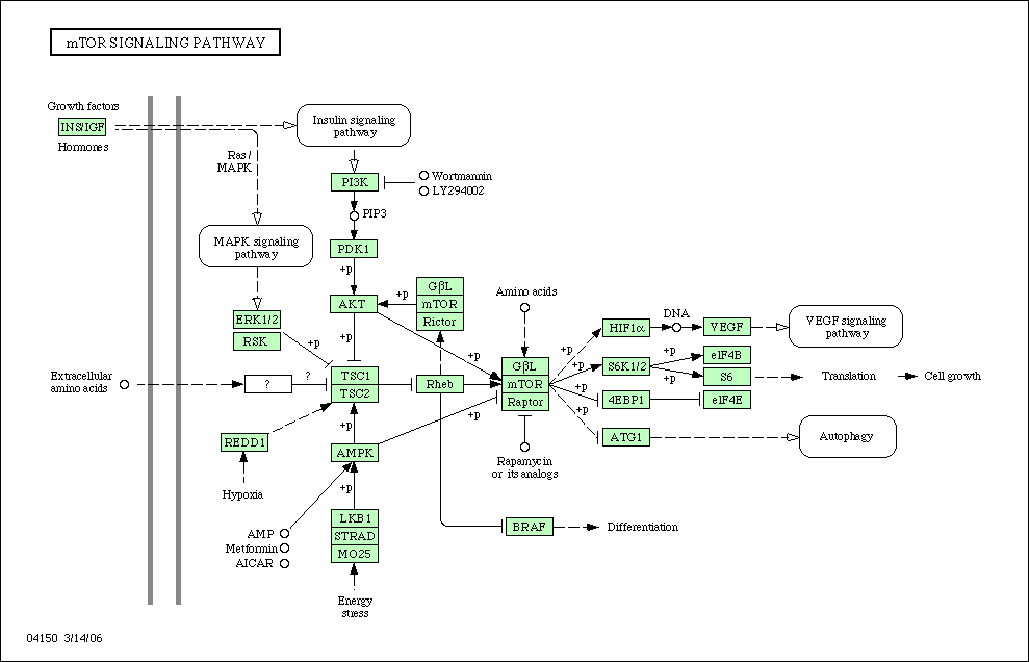

Supplement: Figure S5 — mTOR signaling pathway. Of the 95 transcripts associated with mTOR signaling pathway, 42 genes were up-regulated in our hENSC, and only 5 transcripts were up regulated in OBNSC. (TIF) [file pone.0033542.s005.tif]

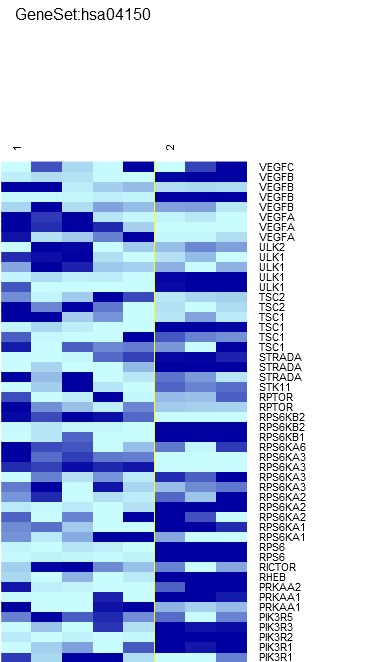

Supplement: Figure S6 — Heat map of the mTOR signaling pathway forming genes in OBNSC Vs. hENSC. (TIF) [file pone.0033542.s006.tif]

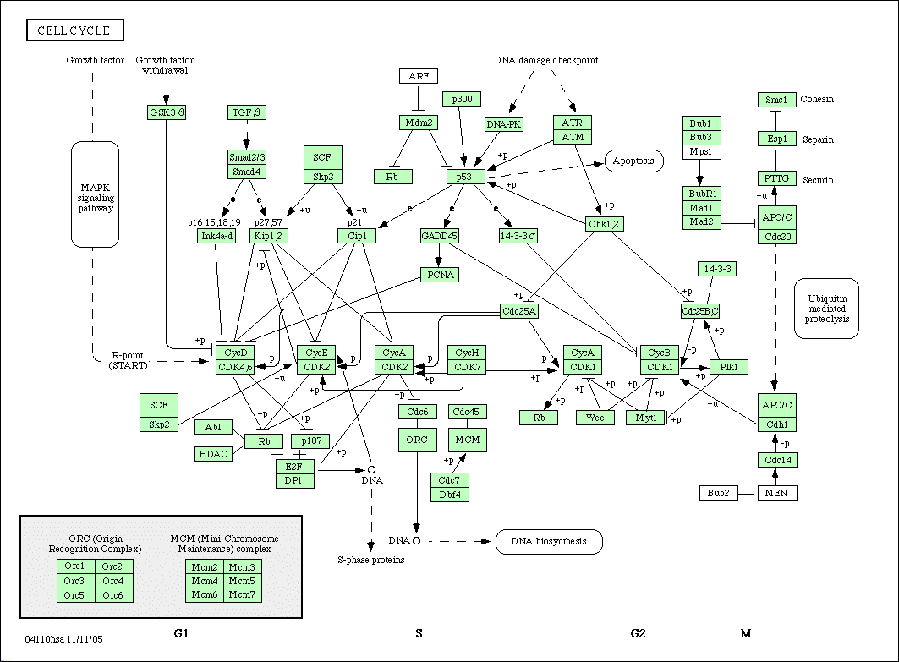

Supplement: Figure S7 — Cell cycle signaling pathway. Gene expression analysis of 200 transcripts of cell cycle signaling molecules revealed the up-regulation of 113 transcripts in hENSC, and only 11 transcripts in OBNSC. (TIF) [file pone.0033542.s007.tif]

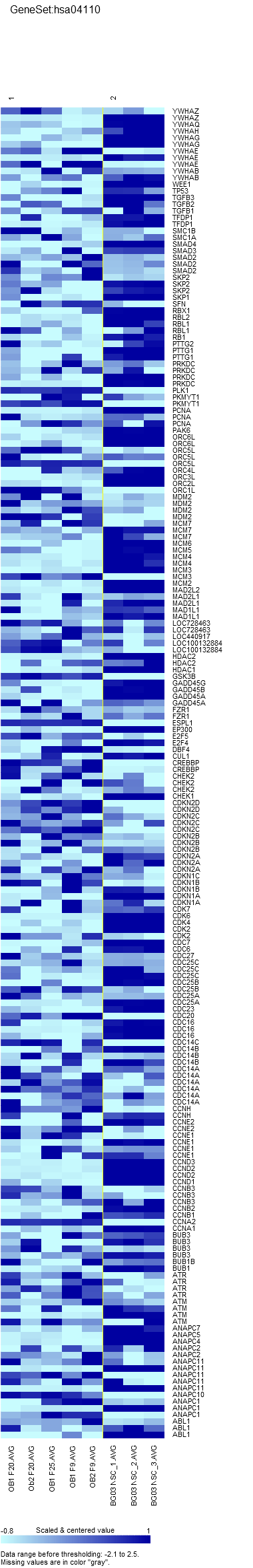

Supplement: Figure S8 — Heat map of the cell cycle signaling pathway forming genes in OBNSC Vs. hENSC. (TIF) [file pone.0033542.s008.tif]

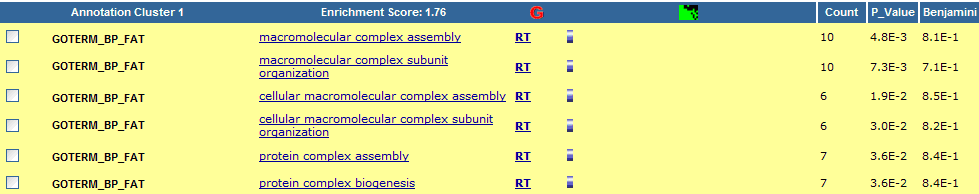

Supplement: Table S6 — Functional Annotation Clustering using DAVID for 203 unregulated genes of human hENSC. 61 annotation clusters were obtained. The annotation cluster 1 included genes related to ribonucleoprotein (19 genes), cytosolic ribosome (13 genes), ribosomal protein (16 genes), translational elongation (13 genes), protein biosynthesis (13 genes), with an enrichment score of 6.78. (TIF) [file pone.0033542.s014.tif]

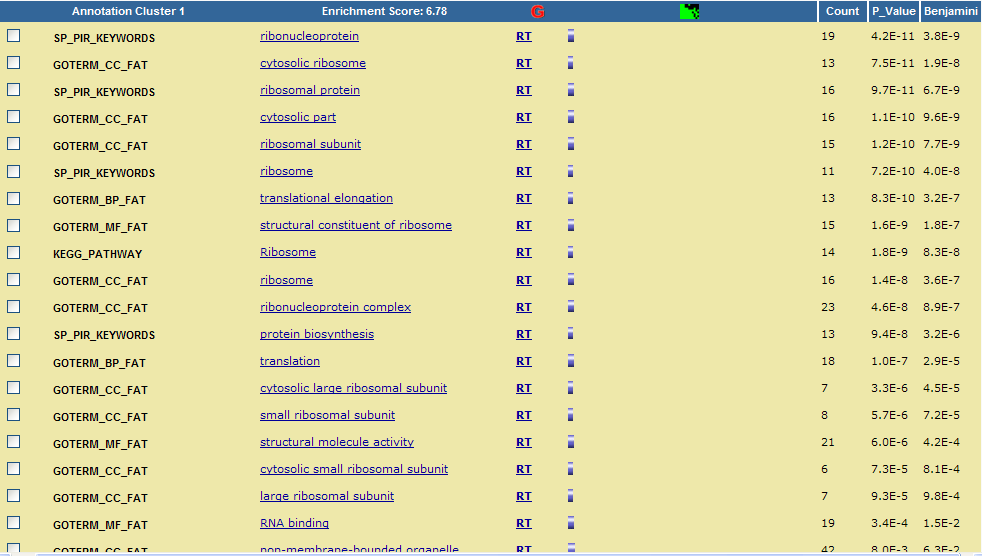

Supplement: Table S7 — Functional Annotation Clustering using DAVID for 203 unregulated genes (Log2≤5–12) of human OBNSC. 28 annotation clusters were obtained. The annotation cluster one included genes related to macromolecular complex assembly (10 genes), macromolecular complex subunit organization (10 genes), cellular macromolecular complex assembly (6 genes), cellular macromolecular complex subunit organization (6 genes), protein complex assembly (7 genes), and protein complex biogenesis (7 genes) with an enrichment score of 27.56. (TIF) [file pone.0033542.s015.tif]
