# Supplementary material for: Gene Expression Profile of Adult Human Olfactory Bulb and Embryonic Neural Stem Cell Suggests Distinct Signaling Pathways and Epigenetic Control
Source: PLoS One. 2012 Apr 2;7(4):e33542. doi: 10.1371/journal.pone.0033542 (PMC3317670; doi:10.1371/journal.pone.0033542)
Supplement: Table S1 — Details of Embryonic and adult NSC samples used in the current study. (DOC) [file pone.0033542.s009.doc]

Supplementary Table S1. Embryonic and adult NSC used in the current study.

| **#** | **Sample**  **index** | **Category** | **Reference** | **Source** |
| --- | --- | --- | --- | --- |
| 1 | ES_NSC1 | ES_NSC | H9 (WA09)^26^ | Invitrogen |
| 2 | ES_NSC2 | ES_NSC | H9 (WA09) ^26^ | Invitrogen |
| 3 | ES_NSC3 | ES_NSC | H9 (WA09) ^26^ | Invitrogen |
| 4 | ES_NSC4 | ES_NSC | H9 (WA09) ^26^ | Invitrogen |
| 5 | ES_NSC5 | ES_NSC | H9 (WA09) ^26^ | Invitrogen |

b. Human Adult Olfactory Bulb Neural Stem Cells

| Sample  index | Age/Sex |  | Diagnosis | Reference | Source |
| --- | --- | --- | --- | --- | --- |
| OB1 |  | Category |  |  |  |
| 1 | 45/M | OB1 P9 | Olfactory groove meningioma | OB1^27^ | Cath. Univ., School of Medicine, Rome, Italy |
| 2 | 45/M | OB1 P20 | Olfactory groove meningioma | OB1 ^27^ | Cath. Univ., School of Medicine, Rome, Italy |
| 3 | 45/M | OB1 P25 | Olfactory groove meningioma | OB1 ^27^ | Cath. Univ., School of Medicine, Rome, Italy |
| OB2 |  |  |  |  |  |
| 1 | 39/M | OB2 P9 | Anterior comunicating artery aneurysm | OB1 ^27^ | Cath. Univ., School of Medicine, Rome, Italy |
| 2 | 39/M | Ob2 P20 | Anterior comunicating artery aneurysm | OB1 ^27^ | Cath. Univ., School of Medicine, Rome, Italy |
| 3 | 39/M | OB2 P22 | Anterior comunicating artery aneurysm | OB1 ^27^ | Cath. Univ., School of Medicine, Rome, Italy |

ES_NSC, ES_NSC, hESC-derived NSC; OB, Olfactory Bulb.
