# Supplementary material for: Gene Expression Profile of Adult Human Olfactory Bulb and Embryonic Neural Stem Cell Suggests Distinct Signaling Pathways and Epigenetic Control
Source: PLoS One. 2012 Apr 2;7(4):e33542. doi: 10.1371/journal.pone.0033542 (PMC3317670; doi:10.1371/journal.pone.0033542)
Supplement: Table S8 — Gene Ontology of differentially expressed transcripts in OBNSCs and hENSC. 25 out of 3875 investigated gene sets passed the 0.005 significance threshold LS/KS permutation test found 125 significant gene sets. Efron-Tibshirani’s maxmean test found 240 significant gene sets (under 200 permutations). By Gene Ontologies: 41 out of the 307 investigated Cellular Component (CC) categories are significant. 45 out of the 620 investigated Molecular Function (MF) categories are significant. 239 out of the 2948 investigated Biological Process (BP) categories are significant. (HTML) [file pone.0033542.s016.html]

 

 
 
 Gene Set Class Comparison Results 
 
P {font-size: 9pt}
TABLE {font-size: 9pt}
 
  
 
 
 
function popUp(text) {
   var prop =
   "location=no,scrollbars=yes,menubars=no,toolbars=no,resizable=yes";
   popup = window.open("","mywin",prop);
   popup.document.open();
   popup.document.write(text);
   popup.document.close();
   popup.focus();
   }
    
 HELP   
   Description of the problem:   
 
Number of classes: 2 
 Column of the Experiment Descriptors sheet that defines class variable: OBNSC vs heNSC
 
Number of genes that passed filtering criteria: 18037
 
Type of Gene Sets: Gene Ontology
 
Number of total investigated Gene Sets: 3875
 
Type of univariate test used:  Two-sample T-test
 Random variance model was not used because distribution assumptions of this model were not satisfied.
 
 
Tests used to find significant gene sets are: LS/KS permutation test, Efron-Tibshirani's GSA maxmean test
 
The threshold of determining significant gene sets is 0.005
 
LS/KS permutation test finds gene sets which have more genes differentially expressed  among the phenotype classes  than expected by chance.
 
Efron-Tibshirani's test uses 'maxmean' statistics to identify gene sets differentially expressed. 


 
 
  Summary of Results:     325  out of  3875  investigated gene sets passed the  0.005  significance threshold    LS/KS permutation test found 125 significant gene sets.   Efron-Tibshirani's maxmean test found 240 significant gene sets (under 200 permutations).     By Gene Ontologies:      41  out of the 307  investigated Cellular Component (CC) categories  are   significant.      45  out of the 620  investigated Molecular Function (MF) categories  are   significant.      239  out of the 2948  investigated Biological Process (BP) categories  are   significant.      
  Table  - Table of Gene Sets: 
325
 gene sets sorted by LS permutation p-value (significant p-values are in red)
 To access the list of genes within each gene set, click the hyperlinked number of genes for each gene set.
 
class 1:1, class 2:2
 
 

 
     GO category GO ontology GO term Number of  genes  Heatmap link LS permutation  p-value  KS permutation  p-value  Efron-Tibshirani's GSA test  p-value 
   1 GO:0051351 BP positive regulation of ligase activity  90   heatmap    0.00001     0.00012      
   2 GO:0051443 BP positive regulation of ubiquitin-protein ligase activity  88   heatmap    0.00001     0.00008      
   3 GO:0000502 CC proteasome complex  82   heatmap    0.00001     0.0002      
   4 GO:0005746 CC mitochondrial respiratory chain  64   heatmap    0.00001     0.00001   0.01 (+)
   5 GO:0015934 CC large ribosomal subunit  74   heatmap    0.00001     0.00001      
   6 GO:0015935 CC small ribosomal subunit  73   heatmap    0.00001     0.00001      
   7 GO:0070469 CC respiratory chain  67   heatmap    0.00001     0.00001      
   8 GO:0015078 MF hydrogen ion transmembrane transporter activity  88   heatmap    0.00001     0.00001      
   9 GO:0051439 BP regulation of ubiquitin-protein ligase activity involved in mitotic cell cycle  86   heatmap    0.00001     0.00033   0.02 (+)
   10 GO:0045737 BP positive regulation of cyclin-dependent protein kinase activity  14   heatmap    0.00002     0.00188   0.02 (+)
   11 GO:0016469 CC proton-transporting two-sector ATPase complex  56   heatmap    0.00003     0.00003   0.025 (+)
   12 GO:0051436 BP negative regulation of ubiquitin-protein ligase activity involved in mitotic cell cycle  81   heatmap    0.00004     0.00048   0.02 (+)
   13 GO:0046930 CC pore complex  84   heatmap    0.00005     0.00003   0.25 (+)
   14 GO:0031145 BP anaphase-promoting complex-dependent proteasomal ubiquitin-dependent protein catabolic process  81   heatmap    0.00005     0.00116   0.02 (+)
   15 GO:0006220 BP pyrimidine nucleotide metabolic process  37   heatmap    0.00008   0.01686 0.095 (+)
   16 GO:0016679 MF oxidoreductase activity, acting on diphenols and related substances as donors  8   heatmap    0.00008     0.00311      
   17 GO:0016681 MF oxidoreductase activity, acting on diphenols and related substances as donors, cytochrome as acceptor  8   heatmap    0.00008     0.00311      
   18 GO:0045275 CC respiratory chain complex III  6   heatmap    0.00008     0.0001      
   19 GO:0022904 BP respiratory electron transport chain  63   heatmap    0.00011     0.00027      
   20 GO:0033178 CC proton-transporting two-sector ATPase complex, catalytic domain  21   heatmap    0.00014     0.00481      
   21 GO:0005643 CC nuclear pore  75   heatmap    0.00015     0.00006   0.285 (+)
   22 GO:0006413 BP translational initiation  66   heatmap    0.00015     0.0001   0.06 (+)
   23 GO:0015985 BP energy coupled proton transport, down electrochemical gradient  49   heatmap    0.0002     0.00016   0.025 (+)
   24 GO:0015986 BP ATP synthesis coupled proton transport  49   heatmap    0.0002     0.00016   0.025 (+)
   25 GO:0045259 CC proton-transporting ATP synthase complex  33   heatmap    0.00021     0.00069   0.04 (+)
   26 GO:0015992 BP proton transport  67   heatmap    0.00029     0.0001   0.025 (+)
   27 GO:0042773 BP ATP synthesis coupled electron transport  52   heatmap    0.00029     0.00008   0.025 (+)
   28 GO:0042775 BP mitochondrial ATP synthesis coupled electron transport  52   heatmap    0.00029     0.00008   0.025 (+)
   29 GO:0000377 BP RNA splicing, via transesterification reactions with bulged adenosine as nucleophile  95   heatmap    0.00029   0.01897 0.11 (+)
   30 GO:0000398 BP nuclear mRNA splicing, via spliceosome  95   heatmap    0.00029   0.01897 0.11 (+)
   31 GO:0016126 BP sterol biosynthetic process  48   heatmap    0.00033   0.01875    
   32 GO:0051352 BP negative regulation of ligase activity  87   heatmap    0.00033     0.00242   0.02 (+)
   33 GO:0051444 BP negative regulation of ubiquitin-protein ligase activity  87   heatmap    0.00033     0.00242   0.02 (+)
   34 GO:0016655 MF oxidoreductase activity, acting on NADH or NADPH, quinone or similar compound as acceptor  45   heatmap    0.00038     0.00029   0.01 (+)
   35 GO:0019829 MF cation-transporting ATPase activity  41   heatmap    0.00044     0.00089      
   36 GO:0030964 CC NADH dehydrogenase complex  38   heatmap    0.00044     0.00008   0.01 (+)
   37 GO:0045271 CC respiratory chain complex I  38   heatmap    0.00044     0.00008   0.01 (+)
   38 GO:0022618 BP ribonucleoprotein complex assembly  72   heatmap    0.00044   0.01429 0.075 (+)
   39 GO:0008173 MF RNA methyltransferase activity  18   heatmap    0.00046     0.00427   0.105 (+)
   40 GO:0000079 BP regulation of cyclin-dependent protein kinase activity  66   heatmap    0.0005     0.00467   0.115 (+)
   41 GO:0009219 BP pyrimidine deoxyribonucleotide metabolic process  11   heatmap    0.00055   0.16801    
   42 GO:0006446 BP regulation of translational initiation  47   heatmap    0.00058     0.00176   0.035 (+)
   43 GO:0016651 MF oxidoreductase activity, acting on NADH or NADPH  73   heatmap    0.00066     0.00269      
   44 GO:0007032 BP endosome organization  18   heatmap    0.00067   0.01644 0.175 (+)
   45 GO:0005753 CC mitochondrial proton-transporting ATP synthase complex  28   heatmap    0.00068     0.00115   0.04 (+)
   46 GO:0015002 MF heme-copper terminal oxidase activity  19   heatmap    0.00072   0.01173    
   47 GO:0016675 MF oxidoreductase activity, acting on heme group of donors  19   heatmap    0.00072   0.01173    
   48 GO:0016676 MF oxidoreductase activity, acting on heme group of donors, oxygen as acceptor  19   heatmap    0.00072   0.01173    
   49 GO:0031397 BP negative regulation of protein ubiquitination  100   heatmap    0.00073     0.00484   0.02 (+)
   50 GO:0005732 CC small nucleolar ribonucleoprotein complex  22   heatmap    0.00073     0.0013   0.075 (+)
   51 GO:0005775 CC vacuolar lumen  6   heatmap    0.00078     0.00087      
   52 GO:0006839 BP mitochondrial transport  84   heatmap    0.00085     0.001      
   53 GO:0016090 BP prenol metabolic process  7   heatmap    0.00088   0.0279 0.015 (+)
   54 GO:0016093 BP polyprenol metabolic process  7   heatmap    0.00088   0.0279 0.015 (+)
   55 GO:0006733 BP oxidoreduction coenzyme metabolic process  47   heatmap    0.00093   0.00545 0.07 (+)
   56 GO:0006818 BP hydrogen transport  70   heatmap    0.001     0.00035   0.025 (+)
   57 GO:0016585 CC chromatin remodeling complex  84   heatmap    0.00103   0.0058 0.17 (+)
   58 GO:0006754 BP ATP biosynthetic process  96   heatmap    0.00104     0.00007   0.025 (+)
   59 GO:0000313 CC organellar ribosome  36   heatmap    0.00104     0.00181   0.14 (+)
   60 GO:0005761 CC mitochondrial ribosome  36   heatmap    0.00104     0.00181   0.14 (+)
   61 GO:0009120 BP deoxyribonucleoside metabolic process  6   heatmap    0.00106   0.05484 0.02 (+)
   62 GO:0003954 MF NADH dehydrogenase activity  37   heatmap    0.00108     0.00011   0.035 (+)
   63 GO:0050136 MF NADH dehydrogenase (quinone) activity  37   heatmap    0.00108     0.00011   0.035 (+)
   64 GO:0016407 MF acetyltransferase activity  67   heatmap    0.00117   0.00643 0.09 (+)
   65 GO:0043297 BP apical junction assembly  5   heatmap    0.00129   0.00673    
   66 GO:0046496 BP nicotinamide nucleotide metabolic process  34   heatmap    0.0013   0.00595 0.04 (+)
   67 GO:0006626 BP protein targeting to mitochondrion  35   heatmap    0.00144     0.00008   0.13 (+)
   68 GO:0070585 BP protein localization in mitochondrion  35   heatmap    0.00144     0.00008   0.13 (+)
   69 GO:0005793 CC ER-Golgi intermediate compartment  54   heatmap    0.00149     0.00207   0.15 (+)
   70 GO:0015036 MF disulfide oxidoreductase activity  20   heatmap    0.00153   0.01039 0.035 (+)
   71 GO:0016860 MF intramolecular oxidoreductase activity  36   heatmap    0.00157   0.01254 0.085 (+)
   72 GO:0000123 CC histone acetyltransferase complex  59   heatmap    0.00171   0.0164 0.055 (-)
   73 GO:0034220 BP ion transmembrane transport  89   heatmap    0.0018     0.00043      
   74 GO:0031902 CC late endosome membrane  57   heatmap    0.0018   0.04378 0.19 (+)
   75 GO:0022624 CC proteasome accessory complex  17   heatmap    0.0018   0.01149 0.035 (+)
   76 GO:0009451 BP RNA modification  48   heatmap    0.00187     0.0019   0.145 (+)
   77 GO:0010340 MF carboxyl-O-methyltransferase activity  7   heatmap    0.00192   0.00614 0.06 (+)
   78 GO:0051998 MF protein carboxyl O-methyltransferase activity  7   heatmap    0.00192   0.00614 0.06 (+)
   79 GO:0009060 BP aerobic respiration  44   heatmap    0.00197   0.02884 0.205 (+)
   80 GO:0008757 MF S-adenosylmethionine-dependent methyltransferase activity  84   heatmap    0.00199   0.0064 0.155 (+)
   81 GO:0005839 CC proteasome core complex  24   heatmap    0.00222   0.00961    
   82 GO:0000307 CC cyclin-dependent protein kinase holoenzyme complex  20   heatmap    0.00225   0.03801    
   83 GO:0042274 BP ribosomal small subunit biogenesis  19   heatmap    0.00227   0.12176   0.005 (+)  
   84 GO:0010923 BP negative regulation of phosphatase activity  9   heatmap    0.00227   0.02141    
   85 GO:0032515 BP negative regulation of phosphoprotein phosphatase activity  9   heatmap    0.00227   0.02141    
   86 GO:0009262 BP deoxyribonucleotide metabolic process  33   heatmap    0.0023   0.3103 0.05 (+)
   87 GO:0016591 CC DNA-directed RNA polymerase II, holoenzyme  75   heatmap    0.00259     0.00084   0.12 (+)
   88 GO:0016667 MF oxidoreductase activity, acting on sulfur group of donors  41   heatmap    0.0026   0.01797 0.035 (+)
   89 GO:0000314 CC organellar small ribosomal subunit  12   heatmap    0.00267   0.0215 0.125 (+)
   90 GO:0019362 BP pyridine nucleotide metabolic process  36   heatmap    0.00272   0.01139 0.04 (+)
   91 GO:0006477 BP protein amino acid sulfation  5   heatmap    0.00277   0.08207 0.085 (+)
   92 GO:0070938 CC contractile ring  5   heatmap    0.00297   0.01177 0.015 (+)
   93 GO:0043666 BP regulation of phosphoprotein phosphatase activity  17   heatmap    0.00305   0.03412 0.155 (+)
   94 GO:0000118 CC histone deacetylase complex  42   heatmap    0.00332   0.01537 0.07 (+)
   95 GO:0051149 BP positive regulation of muscle cell differentiation  16   heatmap    0.00342   0.00842    
   96 GO:0006275 BP regulation of DNA replication  54   heatmap    0.00356   0.09182 0.09 (+)
   97 GO:0042625 MF ATPase activity, coupled to transmembrane movement of ions  68   heatmap    0.00382     0.00039      
   98 GO:0009086 BP methionine biosynthetic process  9   heatmap    0.00417   0.03637    
   99 GO:0008080 MF N-acetyltransferase activity  57   heatmap    0.00433   0.02722 0.26 (+)
   100 GO:0009116 BP nucleoside metabolic process  65   heatmap    0.00437   0.00565 0.16 (+)
   101 GO:0016574 BP histone ubiquitination  13   heatmap    0.00457   0.1326 0.01 (+)
   102 GO:0006984 BP ER-nucleus signaling pathway  34   heatmap    0.00462   0.15113 0.18 (+)
   103 GO:0046356 BP acetyl-CoA catabolic process  29   heatmap    0.00474   0.02293 0.22 (+)
   104 GO:0045261 CC proton-transporting ATP synthase complex, catalytic core F(1)  10   heatmap    0.00477   0.02584 0.025 (+)
   105 GO:0008033 BP tRNA processing  72   heatmap    0.00484     0.00132   0.165 (+)
   106 GO:0006352 BP transcription initiation  89   heatmap    0.00487     0.00134   0.085 (-)
   107 GO:0048806 BP genitalia development  17   heatmap  0.00518 0.06274    
   108 GO:0003729 MF mRNA binding  72   heatmap  0.00584   0.00158      
   109 GO:0048144 BP fibroblast proliferation  38   heatmap  0.00627 0.07128    
   110 GO:0048145 BP regulation of fibroblast proliferation  38   heatmap  0.00627 0.07128    
   111 GO:0009067 BP aspartate family amino acid biosynthetic process  17   heatmap  0.00657 0.02681    
   112 GO:0006555 BP methionine metabolic process  13   heatmap  0.00746 0.00773    
   113 GO:0033176 CC proton-transporting V-type ATPase complex  19   heatmap  0.00805   0.0038      
   114 GO:0019348 BP dolichol metabolic process  5   heatmap  0.00866 0.0878    
   115 GO:0042026 BP protein refolding  16   heatmap  0.00949 0.04918    
   116 GO:0016638 MF oxidoreductase activity, acting on the CH-NH2 group of donors  8   heatmap  0.0095 0.04943    
   117 GO:0006694 BP steroid biosynthetic process  83   heatmap  0.0098 0.02274    
   118 GO:0006695 BP cholesterol biosynthetic process  35   heatmap  0.01014 0.06254    
   119 GO:0045445 BP myoblast differentiation  26   heatmap  0.01091 0.01034    
   120 GO:0005838 CC proteasome regulatory particle  13   heatmap  0.01153 0.00948    
   121 GO:0006611 BP protein export from nucleus  26   heatmap  0.01187 0.00966    
   122 GO:0016251 MF general RNA polymerase II transcription factor activity  48   heatmap  0.01436 0.00909    
   123 GO:0009066 BP aspartate family amino acid metabolic process  23   heatmap  0.01625 0.0166    
   124 GO:0060323 BP head morphogenesis  13   heatmap  0.01637 0.03273    
   125 GO:0043189 CC H4/H2A histone acetyltransferase complex  24   heatmap  0.01656 0.06782    
   126 GO:0043516 BP regulation of DNA damage response, signal transduction by p53 class mediator  14   heatmap  0.01729 0.01876    
   127 GO:0000380 BP alternative nuclear mRNA splicing, via spliceosome  12   heatmap  0.01738   0.00067   0.515 (-)
   128 GO:0032182 MF small conjugating protein binding  39   heatmap  0.01908   0.00353   0.14 (+)
   129 GO:0051427 MF hormone receptor binding  96   heatmap  0.02009   0.0009      
   130 GO:0006999 BP nuclear pore organization  8   heatmap  0.02164   0.00102   0.015 (-)
   131 GO:0006596 BP polyamine biosynthetic process  9   heatmap  0.02296 0.03714    
   132 GO:0031122 BP cytoplasmic microtubule organization  7   heatmap  0.02351 0.12196    
   133 GO:0006734 BP NADH metabolic process  8   heatmap  0.02456 0.26853    
   134 GO:0051147 BP regulation of muscle cell differentiation  31   heatmap  0.02589 0.03316    
   135 GO:0048199 BP vesicle targeting, to, from or within Golgi  14   heatmap  0.02633   0.00195   0.065 (+)
   136 GO:0001836 BP release of cytochrome c from mitochondria  32   heatmap  0.02636 0.17571    
   137 GO:0035140 BP arm morphogenesis  5   heatmap  0.02788 0.07926    
   138 GO:0071680 BP response to indole-3-methanol  9   heatmap  0.02792 0.28239    
   139 GO:0042303 BP molting cycle  28   heatmap  0.02837 0.33948    
   140 GO:0042633 BP hair cycle  28   heatmap  0.02837 0.33948    
   141 GO:0043030 BP regulation of macrophage activation  9   heatmap  0.02869 0.00558    
   142 GO:0060343 BP trabecula formation  11   heatmap  0.02927 0.06767    
   143 GO:0003727 MF single-stranded RNA binding  35   heatmap  0.0303 0.0251    
   144 GO:0014009 BP glial cell proliferation  5   heatmap  0.03205 0.07926    
   145 GO:0014010 BP Schwann cell proliferation  5   heatmap  0.03205 0.07926    
   146 GO:0051153 BP regulation of striated muscle cell differentiation  27   heatmap  0.03224 0.0424    
   147 GO:0019211 MF phosphatase activator activity  5   heatmap  0.03295 0.10683    
   148 GO:0050919 BP negative chemotaxis  12   heatmap  0.03349 0.40324    
   149 GO:0016820 MF hydrolase activity, acting on acid anhydrides, catalyzing transmembrane movement of substances  95   heatmap  0.03411   0.00237      
   150 GO:0031057 BP negative regulation of histone modification  7   heatmap  0.03486 0.12501    
   151 GO:0051155 BP positive regulation of striated muscle cell differentiation  7   heatmap  0.03592 0.12189    
   152 GO:0035257 MF nuclear hormone receptor binding  87   heatmap  0.03629   0.00318      
   153 GO:0009948 BP anterior/posterior axis specification  13   heatmap  0.03632 0.0659    
   154 GO:0008299 BP isoprenoid biosynthetic process  20   heatmap  0.03695 0.45465    
   155 GO:0042987 BP amyloid precursor protein catabolic process  17   heatmap  0.03724 0.25964    
   156 GO:0008637 BP apoptotic mitochondrial changes  42   heatmap  0.03858 0.12791    
   157 GO:0043492 MF ATPase activity, coupled to movement of substances  94   heatmap  0.03961   0.00302      
   158 GO:0046912 MF transferase activity, transferring acyl groups, acyl groups converted into alkyl on transfer  7   heatmap  0.04017 0.12402    
   159 GO:0032512 BP regulation of protein phosphatase type 2B activity  5   heatmap  0.04047 0.10254    
   160 GO:0061041 BP regulation of wound healing  35   heatmap  0.04103 0.04459    
   161 GO:0042626 MF ATPase activity, coupled to transmembrane movement of substances  93   heatmap  0.04143   0.00394      
   162 GO:0071780 BP mitotic cell cycle G2/M transition checkpoint  5   heatmap  0.04196 0.07523    
   163 GO:0060431 BP primary lung bud formation  5   heatmap  0.04562 0.39288    
   164 GO:0032351 BP negative regulation of hormone metabolic process  5   heatmap  0.04716 0.05829    
   165 GO:0032353 BP negative regulation of hormone biosynthetic process  5   heatmap  0.04716 0.05829    
   166 GO:0090032 BP negative regulation of steroid hormone biosynthetic process  5   heatmap  0.04716 0.05829    
   167 GO:0051920 MF peroxiredoxin activity  11   heatmap  0.04752   0.00199   0.05 (+)
   168 GO:0007063 BP regulation of sister chromatid cohesion  8   heatmap  0.04841 0.33079    
   169 GO:0046825 BP regulation of protein export from nucleus  10   heatmap  0.0492 0.14884    
   170 GO:0005902 CC microvillus  37   heatmap  0.0496 0.49412    
   171 GO:0035112 BP genitalia morphogenesis  5   heatmap  0.05041 0.39288    
   172 GO:0031528 CC microvillus membrane  13   heatmap  0.05061 0.21041    
   173 GO:0019843 MF rRNA binding  31   heatmap  0.05073 0.08516    
   174 GO:0016717 MF oxidoreductase activity, acting on paired donors, with oxidation of a pair of donors resulting in the reduction of molecular oxygen to two molecules of water  5   heatmap  0.05153 0.22061    
   175 GO:0006390 BP transcription from mitochondrial promoter  7   heatmap  0.05208 0.29012    
   176 GO:0010894 BP negative regulation of steroid biosynthetic process  9   heatmap  0.05267   0.00485      
   177 GO:0045939 BP negative regulation of steroid metabolic process  9   heatmap  0.05267   0.00485      
   178 GO:0048742 BP regulation of skeletal muscle fiber development  23   heatmap  0.05429 0.05616    
   179 GO:0033599 BP regulation of mammary gland epithelial cell proliferation  9   heatmap  0.05573 0.04894    
   180 GO:0050818 BP regulation of coagulation  35   heatmap  0.05778 0.09516    
   181 GO:0001942 BP hair follicle development  27   heatmap  0.05835 0.39117    
   182 GO:0022404 BP molting cycle process  27   heatmap  0.05835 0.39117    
   183 GO:0022405 BP hair cycle process  27   heatmap  0.05835 0.39117    
   184 GO:0009452 BP RNA capping  6   heatmap  0.05847   0.00224   0.14 (+)
   185 GO:0003338 BP metanephros morphogenesis  7   heatmap  0.05871 0.16515    
   186 GO:0030522 BP intracellular receptor mediated signaling pathway  89   heatmap  0.05898 0.02294    
   187 GO:0045995 BP regulation of embryonic development  22   heatmap  0.06043 0.10632    
   188 GO:0050435 BP beta-amyloid metabolic process  11   heatmap  0.06102 0.25829    
   189 GO:0045661 BP regulation of myoblast differentiation  12   heatmap  0.06132 0.0694    
   190 GO:0003401 BP axis elongation  5   heatmap  0.06144 0.20707    
   191 GO:0060602 BP branch elongation of an epithelium  5   heatmap  0.06144 0.20707    
   192 GO:0016579 BP protein deubiquitination  44   heatmap  0.06171 0.03594    
   193 GO:0031576 BP G2/M transition checkpoint  26   heatmap  0.06338 0.16811    
   194 GO:0048008 BP platelet-derived growth factor receptor signaling pathway  24   heatmap  0.0659 0.04764    
   195 GO:0060438 BP trachea development  5   heatmap  0.06888 0.39288    
   196 GO:0060439 BP trachea morphogenesis  5   heatmap  0.06888 0.39288    
   197 GO:0035258 MF steroid hormone receptor binding  55   heatmap  0.0692 0.01856    
   198 GO:0060993 BP kidney morphogenesis  9   heatmap  0.07523 0.07879    
   199 GO:0051055 BP negative regulation of lipid biosynthetic process  20   heatmap  0.07674 0.09659    
   200 GO:0030521 BP androgen receptor signaling pathway  57   heatmap  0.07993 0.02488    
   201 GO:0060688 BP regulation of morphogenesis of a branching structure  17   heatmap  0.08349 0.14779    
   202 GO:0016202 BP regulation of striated muscle tissue development  40   heatmap  0.08828 0.03566    
   203 GO:0004457 MF lactate dehydrogenase activity  5   heatmap  0.08832 0.66734    
   204 GO:0060479 BP lung cell differentiation  8   heatmap  0.0899 0.30171    
   205 GO:0048641 BP regulation of skeletal muscle tissue development  27   heatmap  0.09133 0.0424    
   206 GO:0048286 BP lung alveolus development  21   heatmap  0.09221 0.23357    
   207 GO:0006613 BP cotranslational protein targeting to membrane  16   heatmap  0.09559   0.00343   0.075 (+)
   208 GO:0033180 CC proton-transporting V-type ATPase, V1 domain  7   heatmap  0.09584 0.42313    
   209 GO:0010830 BP regulation of myotube differentiation  6   heatmap  0.09707 0.1675    
   210 GO:0006612 BP protein targeting to membrane  34   heatmap  0.09786   0.0022   0.115 (+)
   211 GO:0070509 BP calcium ion import  6   heatmap  0.09801 0.03804    
   212 GO:0070588 BP calcium ion transmembrane transport  6   heatmap  0.09801 0.03804    
   213 GO:0090279 BP regulation of calcium ion import  6   heatmap  0.09801 0.03804    
   214 GO:0030518 BP steroid hormone receptor signaling pathway  76   heatmap  0.09954 0.02674    
   215 GO:0071363 BP cellular response to growth factor stimulus  18   heatmap  0.10205 0.2217    
   216 GO:0015662 MF ATPase activity, coupled to transmembrane movement of ions, phosphorylative mechanism  48   heatmap  0.10304 0.00878    
   217 GO:0006720 BP isoprenoid metabolic process  40   heatmap  0.1131 0.82238    
   218 GO:0001656 BP metanephros development  27   heatmap  0.11395 0.14719    
   219 GO:0001706 BP endoderm formation  5   heatmap  0.11527 0.39288    
   220 GO:0031229 CC intrinsic to nuclear inner membrane  5   heatmap  0.11543 0.31224    
   221 GO:0010559 BP regulation of glycoprotein biosynthetic process  16   heatmap  0.11629 0.22102    
   222 GO:0060571 BP morphogenesis of an epithelial fold  11   heatmap  0.11659 0.2482    
   223 GO:0008203 BP cholesterol metabolic process  79   heatmap  0.11835 0.21018    
   224 GO:0004869 MF cysteine-type endopeptidase inhibitor activity  23   heatmap  0.11998   0.00496   0.06 (+)
   225 GO:0048634 BP regulation of muscle organ development  41   heatmap  0.12393 0.05916    
   226 GO:0032653 BP regulation of interleukin-10 production  6   heatmap  0.12611 0.16442    
   227 GO:0060572 BP morphogenesis of an epithelial bud  9   heatmap  0.12675 0.41436    
   228 GO:0014014 BP negative regulation of gliogenesis  8   heatmap  0.12936 0.32699    
   229 GO:0004448 MF isocitrate dehydrogenase activity  7   heatmap  0.13293 0.24204    
   230 GO:0090030 BP regulation of steroid hormone biosynthetic process  6   heatmap  0.14344 0.18172    
   231 GO:0014902 BP myotube differentiation  18   heatmap  0.14349 0.37104    
   232 GO:0043583 BP ear development  56   heatmap  0.14454 0.04058    
   233 GO:0010543 BP regulation of platelet activation  8   heatmap  0.14513 0.60795    
   234 GO:0006661 BP phosphatidylinositol biosynthetic process  7   heatmap  0.15075 0.36525    
   235 GO:0010389 BP regulation of G2/M transition of mitotic cell cycle  8   heatmap  0.1528 0.11854    
   236 GO:0072001 BP renal system development  80   heatmap  0.15892 0.54831    
   237 GO:0014909 BP smooth muscle cell migration  24   heatmap  0.16309 0.08807    
   238 GO:0021781 BP glial cell fate commitment  8   heatmap  0.16756 0.12377    
   239 GO:0045601 BP regulation of endothelial cell differentiation  6   heatmap  0.1699 0.1994    
   240 GO:0010457 BP centriole-centriole cohesion  6   heatmap  0.17076 0.1994    
   241 GO:0014910 BP regulation of smooth muscle cell migration  22   heatmap  0.17279 0.06571    
   242 GO:0007492 BP endoderm development  16   heatmap  0.18332 0.22102    
   243 GO:0055002 BP striated muscle cell development  68   heatmap  0.18826 0.45133    
   244 GO:0010829 BP negative regulation of glucose transport  5   heatmap  0.19047 0.30565    
   245 GO:0001822 BP kidney development  79   heatmap  0.19048 0.58133    
   246 GO:0010464 BP regulation of mesenchymal cell proliferation  18   heatmap  0.19185 0.28565    
   247 GO:0034765 BP regulation of ion transmembrane transport  35   heatmap  0.19195 0.09878    
   248 GO:0060684 BP epithelial-mesenchymal cell signaling  5   heatmap  0.19273 0.53004    
   249 GO:0010463 BP mesenchymal cell proliferation  20   heatmap  0.19568 0.20344    
   250 GO:0022409 BP positive regulation of cell-cell adhesion  14   heatmap  0.19595 0.48582    
   251 GO:0042147 BP retrograde transport, endosome to Golgi  12   heatmap  0.19667 0.41516    
   252 GO:0014704 CC intercalated disc  13   heatmap  0.20422 0.21041    
   253 GO:0044291 CC cell-cell contact zone  13   heatmap  0.20422 0.21041    
   254 GO:0014812 BP muscle cell migration  26   heatmap  0.21023 0.11572    
   255 GO:0031572 BP G2/M transition DNA damage checkpoint  23   heatmap  0.21118 0.29402    
   256 GO:0044453 CC nuclear membrane part  6   heatmap  0.21523 0.45874    
   257 GO:0007440 BP foregut morphogenesis  8   heatmap  0.21978 0.39178    
   258 GO:0001709 BP cell fate determination  20   heatmap  0.22418 0.17748    
   259 GO:0031128 BP developmental induction  17   heatmap  0.22749 0.14779    
   260 GO:0045168 BP cell-cell signaling involved in cell fate commitment  17   heatmap  0.22749 0.14779    
   261 GO:2000027 BP regulation of organ morphogenesis  40   heatmap  0.22982 0.30191    
   262 GO:0022829 MF wide pore channel activity  11   heatmap  0.23239 0.31232    
   263 GO:0034661 BP ncRNA catabolic process  5   heatmap  0.23396 0.16219    
   264 GO:0022011 BP myelination in the peripheral nervous system  7   heatmap  0.24738 0.20028    
   265 GO:0032292 BP ensheathment of axons in the peripheral nervous system  7   heatmap  0.24738 0.20028    
   266 GO:0060795 BP cell fate commitment involved in the formation of primary germ layers  12   heatmap  0.24921 0.53354    
   267 GO:0051148 BP negative regulation of muscle cell differentiation  11   heatmap  0.24931 0.30063    
   268 GO:0032613 BP interleukin-10 production  7   heatmap  0.24968 0.41649    
   269 GO:0003156 BP regulation of organ formation  15   heatmap  0.25035 0.23021    
   270 GO:0008212 BP mineralocorticoid metabolic process  5   heatmap  0.25442 0.79107    
   271 GO:0032341 BP aldosterone metabolic process  5   heatmap  0.25442 0.79107    
   272 GO:0032344 BP regulation of aldosterone metabolic process  5   heatmap  0.25442 0.79107    
   273 GO:0010560 BP positive regulation of glycoprotein biosynthetic process  7   heatmap  0.25582 0.48299    
   274 GO:0048593 BP camera-type eye morphogenesis  44   heatmap  0.25724 0.1595    
   275 GO:0060428 BP lung epithelium development  7   heatmap  0.26376 0.71674    
   276 GO:0021772 BP olfactory bulb development  10   heatmap  0.26838 0.58231    
   277 GO:0070848 BP response to growth factor stimulus  25   heatmap  0.27456 0.36138    
   278 GO:0048546 BP digestive tract morphogenesis  16   heatmap  0.28675 0.42243    
   279 GO:0060445 BP branching involved in salivary gland morphogenesis  13   heatmap  0.28897 0.78534    
   280 GO:0007431 BP salivary gland development  17   heatmap  0.31598 0.63796    
   281 GO:0007435 BP salivary gland morphogenesis  17   heatmap  0.31598 0.63796    
   282 GO:0051591 BP response to cAMP  35   heatmap  0.31942 0.63716    
   283 GO:0060487 BP lung epithelial cell differentiation  5   heatmap  0.32517 0.53004    
   284 GO:0071664 CC catenin-TCF7L2 complex  6   heatmap  0.32573 0.58108    
   285 GO:0030324 BP lung development  84   heatmap  0.3309 0.55397    
   286 GO:0060541 BP respiratory system development  91   heatmap  0.33586 0.57742    
   287 GO:0060767 BP epithelial cell proliferation involved in prostate gland development  8   heatmap  0.33871 0.39178    
   288 GO:0060441 BP epithelial tube branching involved in lung morphogenesis  15   heatmap  0.34198 0.87856    
   289 GO:0048660 BP regulation of smooth muscle cell proliferation  41   heatmap  0.34601 0.56779    
   290 GO:0010092 BP specification of organ identity  11   heatmap  0.34904 0.63918    
   291 GO:0032330 BP regulation of chondrocyte differentiation  11   heatmap  0.3501 0.63918    
   292 GO:0008210 BP estrogen metabolic process  10   heatmap  0.35146 0.41755    
   293 GO:0021988 BP olfactory lobe development  11   heatmap  0.35476 0.61265    
   294 GO:0033057 BP reproductive behavior in a multicellular organism  6   heatmap  0.35715 0.23453    
   295 GO:0005313 MF L-glutamate transmembrane transporter activity  7   heatmap  0.36692 0.74279    
   296 GO:0002065 BP columnar/cuboidal epithelial cell differentiation  6   heatmap  0.37005 0.46469    
   297 GO:0001759 BP induction of an organ  13   heatmap  0.37019 0.35675    
   298 GO:0051924 BP regulation of calcium ion transport  58   heatmap  0.37115 0.15888    
   299 GO:0030323 BP respiratory tube development  87   heatmap  0.38028 0.53857    
   300 GO:0050819 BP negative regulation of coagulation  19   heatmap  0.40609 0.3386    
   301 GO:0015074 BP DNA integration  14   heatmap  0.40974   0.00343   0.185 (+)
   302 GO:0048659 BP smooth muscle cell proliferation  42   heatmap  0.41361 0.64659    
   303 GO:0045670 BP regulation of osteoclast differentiation  20   heatmap  0.43188 0.88755    
   304 GO:0009084 BP glutamine family amino acid biosynthetic process  16   heatmap  0.43618 0.72201    
   305 GO:0030195 BP negative regulation of blood coagulation  18   heatmap  0.43749 0.39532    
   306 GO:0034332 BP adherens junction organization  10   heatmap  0.4823 0.45912    
   307 GO:0060768 BP regulation of epithelial cell proliferation involved in prostate gland development  7   heatmap  0.48245 0.48299    
   308 GO:0030168 BP platelet activation  25   heatmap  0.4838 0.66342    
   309 GO:0006656 BP phosphatidylcholine biosynthetic process  10   heatmap  0.48971 0.63948    
   310 GO:0042762 BP regulation of sulfur metabolic process  6   heatmap  0.49192 0.78499    
   311 GO:0051176 BP positive regulation of sulfur metabolic process  6   heatmap  0.49192 0.78499    
   312 GO:0046890 BP regulation of lipid biosynthetic process  44   heatmap  0.49301 0.34266    
   313 GO:0030316 BP osteoclast differentiation  24   heatmap  0.51522 0.8574    
   314 GO:0016833 MF oxo-acid-lyase activity  8   heatmap  0.52644 0.31491    
   315 GO:0060425 BP lung morphogenesis  18   heatmap  0.55469 0.90111    
   316 GO:0048730 BP epidermis morphogenesis  14   heatmap  0.57303 0.68874    
   317 GO:0035272 BP exocrine system development  20   heatmap  0.60507 0.88143    
   318 GO:0001933 BP negative regulation of protein amino acid phosphorylation  41   heatmap  0.69008 0.86242    
   319 GO:0045638 BP negative regulation of myeloid cell differentiation  23   heatmap  0.74313 0.8285    
   320 GO:0019865 MF immunoglobulin binding  12   heatmap  0.78423 0.89304    
   321 GO:0002762 BP negative regulation of myeloid leukocyte differentiation  12   heatmap  0.79936 0.87454    
   322 GO:0014046 BP dopamine secretion  8   heatmap  0.82474 0.83797    
   323 GO:0014059 BP regulation of dopamine secretion  8   heatmap  0.82474 0.83797    
   324 GO:0050804 BP regulation of synaptic transmission  100   heatmap  0.91574 0.85457    
   325 GO:0050654 BP chondroitin sulfate proteoglycan metabolic process  14   heatmap  0.9278 0.79277    
 
 
 Links to the genes within gene sets: 

 Gene Sets 1 - 100 
 ... 
 Gene Sets 101 - 200 
 ... 
 Gene Sets 201 - 300 
 ... 
 Gene Sets 301 - 325 
 
 
    Filtering parameters:    
R version 2.12.0 (2010-10-15)
 
Name of the project file: Hany_Project.xls
 
Time of the analysis: Fri Sep 30 15:06:38 2011
  
BRB-ArrayTools Version: 4.2.0 - Beta_2  (June 2011)
 
Project annotated by SOURCE (source.stanford.edu), searched by gene identifier: Symbol, on 7/22/2011 3:40:29 PM
 
   Spot Filters: OFF  
  
   Average the replicate spots within an array: OFF
   
    
Normalization: 
   
Normalize (center) each array
using quantile normalization.
 
   Gene Filters: OFF
   
    Gene Subsets: 
 
INCLUDE ONLY genes in any of the following genelists:
      (Analysis:present_plosonedata);       (Analysis:Presenti in tutti OBNSC)   
  
