# Supplementary material for: Gene Expression Profile of Adult Human Olfactory Bulb and Embryonic Neural Stem Cell Suggests Distinct Signaling Pathways and Epigenetic Control
Source: PLoS One. 2012 Apr 2;7(4):e33542. doi: 10.1371/journal.pone.0033542 (PMC3317670; doi:10.1371/journal.pone.0033542)
Supplement: Table S9 — KEGG Pathway of differentially expressed transcripts between OBNSCs and hENSCs. 75 out of 171 investigated gene sets passed the 0.005 significance threshold LS/KS permutation test found 72 significant gene sets. Efron-Tibshirani’s maxmean test found 17 significant gene sets (under 200 permutations). (HTML) [file pone.0033542.s017.html]

 

 
 
 Gene Set Class Comparison Results 
 
P {font-size: 9pt}
TABLE {font-size: 9pt}
 
  
 
 
 
function popUp(text) {
   var prop =
   "location=no,scrollbars=yes,menubars=no,toolbars=no,resizable=yes";
   popup = window.open("","mywin",prop);
   popup.document.open();
   popup.document.write(text);
   popup.document.close();
   popup.focus();
   }
    
 HELP   
   Description of the problem:   
 
Number of classes: 2 
 Column of the Experiment Descriptors sheet that defines class variable: OBNSC vs heNSC
 
Number of genes that passed filtering criteria: 47231
 
Type of Gene Sets: Kegg  Pathway
 
Number of total investigated Gene Sets: 171
 
Type of univariate test used:  Two-sample T-test
 Random variance model was not used because distribution assumptions of this model were not satisfied.
 
 
Tests used to find significant gene sets are: LS/KS permutation test, Efron-Tibshirani's GSA maxmean test
 
The threshold of determining significant gene sets is 0.005
 
LS/KS permutation test finds gene sets which have more genes differentially expressed  among the phenotype classes  than expected by chance.
 
Efron-Tibshirani's test uses 'maxmean' statistics to identify gene sets differentially expressed. 


 
 
  Summary of Results:     75  out of  171  investigated gene sets passed the  0.005  significance threshold    LS/KS permutation test found 72 significant gene sets.   Efron-Tibshirani's maxmean test found 17 significant gene sets (under 200 permutations).       
  Table  - Table of Gene Sets: 
75
 gene sets sorted by LS permutation p-value (significant p-values are in red)
 To access the list of genes within each gene set, click the hyperlinked number of genes for each gene set.
 
class 1:1, class 2:2
 
 

 
     Kegg Pathway Pathway description Number of  genes  Heatmap link LS permutation  p-value  KS permutation  p-value  Efron-Tibshirani's GSA test  p-value 
   1 hsa00010  Glycolysis / Gluconeogenesis   121   heatmap    0.00001   0.09652 0.115 (+)
   2 hsa00020  Citrate cycle (TCA cycle)   49   heatmap    0.00001     0.00164      
   3 hsa00062  Fatty acid elongation in mitochondria   13   heatmap    0.00001     0.00005      
   4 hsa00100  Biosynthesis of steroids   25   heatmap    0.00001     0.00073   0.02 (+)
   5 hsa00190  Oxidative phosphorylation   159   heatmap    0.00001     0.00001      
   6 hsa00193  ATP synthesis   66   heatmap    0.00001     0.00001      
   7 hsa00230  Purine metabolism   258   heatmap    0.00001     0.00046   0.25 (+)
   8 hsa00240  Pyrimidine metabolism   142   heatmap    0.00001     0.00001   0.25 (+)
   9 hsa00280  Valine, leucine and isoleucine degradation   67   heatmap    0.00001     0.00039   0.17 (+)
   10 hsa00380  Tryptophan metabolism   138   heatmap    0.00001     0.00223   0.235 (+)
   11 hsa00640  Propanoate metabolism   62   heatmap    0.00001   0.01057 0.06 (+)
   12 hsa00650  Butanoate metabolism   63   heatmap    0.00001     0.00217   0.12 (+)
   13 hsa00790  Folate biosynthesis   53   heatmap    0.00001     0.00043   0.275 (+)
   14 hsa00900  Terpenoid biosynthesis   9   heatmap    0.00001     0.0004      
   15 hsa00970  Aminoacyl-tRNA biosynthesis   40   heatmap    0.00001     0.00001   0.23 (+)
   16 hsa03010  Ribosome   150   heatmap    0.00001     0.00001      
   17 hsa03050  Proteasome   49   heatmap    0.00001     0.00001      
   18 hsa04110  Cell cycle   200   heatmap    0.00001     0.00001   0.16 (+)
   19 hsa04130  SNARE interactions in vesicular transport   55   heatmap    0.00001     0.00186   0.25 (+)
   20 hsa04510  Focal adhesion   359   heatmap    0.00001     0.0024      
   21 hsa04520  Adherens junction   154   heatmap    0.00001     0.00092      
   22 hsa04720  Long-term potentiation   111   heatmap    0.00001     0.0007      
   23 hsa04810  Regulation of actin cytoskeleton   357   heatmap    0.00001   0.03696 0.155 (+)
   24 hsa05120  Epithelial cell signaling in Helicobacter pylori infection   118   heatmap    0.00001   0.02608 0.09 (-)
   25 hsa05210  Colorectal cancer   148   heatmap    0.00001     0.00002   0.035 (-)
   26 hsa05060  Prion disease   24   heatmap    0.00002     0.00048      
   27 hsa04310  Wnt signaling pathway   269   heatmap    0.00002     0.001   0.045 (-)
   28 hsa00310  Lysine degradation   90   heatmap    0.00003     0.00393   0.235 (+)
   29 hsa00620  Pyruvate metabolism   83   heatmap    0.00003   0.02507 0.07 (+)
   30 hsa04910  Insulin signaling pathway   236   heatmap    0.00003   0.03914 0.195 (-)
   31 hsa00626  Nitrobenzene degradation   23   heatmap    0.00004     0.00009   0.165 (+)
   32 hsa00440  Aminophosphonate metabolism   29   heatmap    0.00004     0.001   0.165 (+)
   33 hsa03020  RNA polymerase   37   heatmap    0.00005   0.00787 0.205 (+)
   34 hsa00030  Pentose phosphate pathway   41   heatmap    0.00006   0.06757 0.045 (+)
   35 hsa00071  Fatty acid metabolism   81   heatmap    0.00007   0.08007 0.035 (+)
   36 hsa00330  Arginine and proline metabolism   85   heatmap    0.00007   0.09745 0.125 (+)
   37 hsa04120  Ubiquitin mediated proteolysis   92   heatmap    0.00007     0.00053   0.41 (+)
   38 hsa00251  Glutamate metabolism   46   heatmap    0.00008   0.07319 0.185 (+)
   39 hsa04360  Axon guidance   229   heatmap    0.00008   0.08966 0.14 (-)
   40 hsa05010  Alzheimer@   47   heatmap    0.00009   0.05598 0.255 (+)
   41 hsa00511  N-Glycan degradation   19   heatmap    0.0001     0.00014   0.345 (+)
   42 hsa00530  Aminosugars metabolism   45   heatmap    0.0001   0.03275 0.155 (-)
   43 hsa01510  Neurodegenerative Disorders   77   heatmap    0.00014   0.07706 0.135 (+)
   44 hsa00340  Histidine metabolism   66   heatmap    0.00015     0.00364   0.25 (+)
   45 hsa03022  Basal transcription factors   53   heatmap    0.00027   0.06649    
   46 hsa05020  Parkinson@   27   heatmap    0.00027   0.00594 0.145 (+)
   47 hsa01032  Glycan structures - degradation   46   heatmap    0.00045     0.00102   0.29 (+)
   48 hsa04150  mTOR signaling pathway   95   heatmap    0.00047   0.27327 0.13 (-)
   49 hsa04540  Gap junction   153   heatmap    0.00059   0.01674 0.145 (-)
   50 hsa04530  Tight junction   225   heatmap    0.0006   0.07017 0.16 (-)
   51 hsa00051  Fructose and mannose metabolism   70   heatmap    0.00091   0.12761 0.3 (+)
   52 hsa00930  Caprolactam degradation   30   heatmap    0.00094   0.09204 0.09 (+)
   53 hsa04010  MAPK signaling pathway   498   heatmap    0.00112   0.6331 0.16 (-)
   54 hsa04710  Circadian rhythm   27   heatmap    0.00131   0.01468 0.06 (-)
   55 hsa03060  Protein export   16   heatmap    0.0014     0.00025   0.175 (+)
   56 hsa00400  Phenylalanine, tyrosine and tryptophan biosynthesis   14   heatmap    0.00145   0.08306 0.075 (+)
   57 hsa00450  Selenoamino acid metabolism   53   heatmap    0.00146   0.01416 0.245 (+)
   58 hsa00290  Valine, leucine and isoleucine biosynthesis   16   heatmap    0.00151   0.00605 0.24 (+)
   59 hsa00410  beta-Alanine metabolism   40   heatmap    0.00168   0.0113 0.12 (+)
   60 hsa00561  Glycerolipid metabolism   102   heatmap    0.00212   0.16484 0.165 (+)
   61 hsa00903  Limonene and pinene degradation   41   heatmap    0.00233   0.04428 0.225 (+)
   62 hsa00532  Chondroitin sulfate biosynthesis   24   heatmap    0.00234     0.0007   0.31 (+)
   63 hsa04670  Leukocyte transendothelial migration   203   heatmap    0.00249   0.62332 0.145 (-)
   64 hsa00710  Carbon fixation   41   heatmap    0.00269   0.14482 0.125 (+)
   65 hsa04330  Notch signaling pathway   81   heatmap    0.0027   0.04124 0.47 (+)
   66 hsa00670  One carbon pool by folate   25   heatmap    0.00274   0.14825 0.215 (+)
   67 hsa00600  Sphingolipid metabolism   65   heatmap    0.00286   0.02053 0.355 (+)
   68 hsa04612  Antigen processing and presentation   141   heatmap    0.00333   0.17877    
   69 hsa00362  Benzoate degradation via hydroxylation   6   heatmap    0.00371   0.01957 0.13 (+)
   70 hsa00510  N-Glycan biosynthesis   62   heatmap    0.004   0.13349 0.35 (+)
   71 hsa00904  Diterpenoid biosynthesis   5   heatmap  0.0102   0.0024      
   72 hsa00052  Galactose metabolism   62   heatmap  0.01908   0.00444   0.385 (+)
   73 hsa00780  Biotin metabolism   14   heatmap  0.05232 0.01355    
   74 hsa05030  Amyotrophic lateral sclerosis (ALS)   32   heatmap  0.10658 0.43079    
   75 hsa00623  2,4-Dichlorobenzoate degradation   6   heatmap  0.1751 0.28288    
 
 
 Links to the genes within gene sets: 

 Gene Sets 1 - 75 
 
 
    Filtering parameters:    
R version 2.12.0 (2010-10-15)
 
Name of the project file: Hany_Project.xls
 
Time of the analysis: Sun Aug 28 12:40:26 2011
  
BRB-ArrayTools Version: 4.2.0 - Beta_2  (June 2011)
 
Project annotated by SOURCE (source.stanford.edu), searched by gene identifier: Symbol, on 7/22/2011 3:40:29 PM
 
   Spot Filters: OFF  
  
   Average the replicate spots within an array: OFF
   
    
Normalization: 
   
Normalize (center) each array
using quantile normalization.
 
   Gene Filters: OFF
   
   Gene Subsets: OFF
   
  
